# Neural Effects of Physical Activity and Movement Interventions in Individuals With Developmental Disabilities–A Systematic Review

**DOI:** 10.3389/fpsyt.2022.794652

**Published:** 2022-02-15

**Authors:** Wan-Chun Su, Nidhi Amonkar, Corina Cleffi, Sudha Srinivasan, Anjana Bhat

**Affiliations:** ^1^Department of Physical Therapy, University of Delaware, Newark, DE, United States; ^2^Biomechanics and Movement Science Program, University of Delaware, Newark, DE, United States; ^3^Physical Therapy Program, Department of Kinesiology, University of Connecticut, Storrs, CT, United States; ^4^Institute for Health, Intervention, and Policy (InCHIP), University of Connecticut, Storrs, CT, United States; ^5^The Connecticut Institute for the Brain and Cognitive Sciences (IBACS), University of Connecticut, Storrs, CT, United States; ^6^Department of Psychological and Brain Sciences, University of Delaware, Newark, DE, United States

**Keywords:** developmental disabilities, autism spectrum disorder, attention-deficit/hyperactivity disorder, movement interventions, exercise, physical activity, neuroimaging, neural effects

## Abstract

Individuals with developmental disabilities present with perceptuo-motor, social communication, and cognitive impairments that often relate to underlying atypical brain structure and functioning. Physical activity/movement interventions improve behavioral performance of individuals with and without developmental disabilities. Majority of the evidence on potential neural mechanisms explaining the impact of physical activity/movement interventions is based on studies in individuals with typical development; there is a dearth of systematic reviews synthesizing the neural effects of physical activity/movement interventions in individuals with developmental disabilities. In this systematic review, we have gathered evidence on the neural effects of physical activity/movement interventions from 32 papers reporting substantial neural effects and behavioral improvements in individuals with developmental disabilities. Chronic intervention effects (multiple sessions) were greater than acute intervention effects (single session). Specifically, using electroencephalogram, functional magnetic resonance imaging, diffusion tensor imaging, and functional near-infrared spectroscopy, studies found physical activity/movement intervention-related changes in neural activity, indicating normalization of cortical arousal in individuals with attention-deficit /hyperactivity disorder (ADHD), increased social brain connectivity in individuals with autism spectrum disorder (ASD), and more efficient executive functioning processes in individuals with a wide range of other developmental disabilities. Despite promising results, more research is clearly needed in this area with larger sample sizes, using standardized neuroimaging tools/variables, and across multiple diagnoses to further explore the neural mechanisms underlying physical activity/movement interventions and to replicate findings from the present review.

## Introduction

Individuals with developmental disabilities, including Autism Spectrum Disorder (ASD), Attention Deficit/Hyperactivity Disorder (ADHD), Developmental Coordination Disorder (DCD), Learning Disabilities (LD), and Intellectual Disabilities (ID), present with multisystem impairments in perceptuo-motor, social communication, and cognitive-behavioral performance, that in turn affects their psychosocial health/well-being and daily functioning (1). In terms of perceptuo-motor impairments, individuals with developmental disabilities present with sensory processing issues as well as motor incoordination/developmental dyspraxia, poor imitation, poor balance, and problems in functional movements such as reaching, walking, and joint actions (2–10). These difficulties could begin early on in life, affect a child's movement exploration of the environment (i.e., through interactions with objects and caregivers), and will have cascading negative effects on other developmental domains (social communication and cognitive) as well as brain structure/functioning (11–23). Individuals with developmental disabilities may also present with difficulties in social communication skills which affects their well-being, daily functioning, and their ability to establish/maintain relationships with peers and caregivers (1, 5, 24–29). They may also have cognitive impairments, such as impaired executive functioning, including poor motor planning, working memory, inhibitory control, and mental flexibility, all of which affect their daily functioning and academic performance (30, 31). Besides the difficulties in different developmental domains, these populations also have lower physical activity levels and are at greater risk of developing obesity (32–35). Physical activity/motor performance is known to have cascading effects on psychosocial well-being and cognitive performance, with low physical activity levels hindering further social and cognitive development in individuals with and without developmental disabilities (36–39). While there are some papers describing potential neural mechanisms of physical activity/movement interventions in healthy populations (40–42), there is a lack of synthesis of neural effects of such interventions in individuals with developmental disabilities. Therefore, this systematic review will focus on identifying the different neuroimaging tools and related biomarkers that objectively assess neural effects of physical activity/movement interventions in individuals with developmental disabilities.

Multiple studies using a single bout of physical activity and/or a longer period of movement interventions reported positive acute (after a single session) and chronic (following multiple sessions) effects on aerobic capacity, gross motor, psychosocial, and cognitive performance in individuals with developmental disabilities (43–45). For example, a meta-analysis of randomized control trials (RCT) conducted in children with ADHD found that physical activity reduced ADHD symptoms (i.e., attention, hyperactivity, impulsivity), anxiety, as well as improved executive functioning and social performance (43). Similarly, a meta-analysis involving children with ID found that acute and chronic physical activity/movement interventions help improve physical (health i.e., cardiovascular health, motor skill, muscular strength, etc.), psychological health (i.e., self-esteem, well-being, social-emotional skills, etc.), and cognitive performance (45). Multiple studies in children with ASD have used whole-body, creative movement therapies, such as music, dance, yoga, theater, and martial arts, in addressing their multisystem impairments (35, 46–56). Our own research group recently conducted a comprehensive review of the effects of creative movement interventions on multisystem performance in children with ASD and found medium-to-large-sized improvements in social communication skills following music and martial arts training, and in motor and cognitive skills following yoga and martial arts training (57). Taken together, physical activity and movement interventions led to positive effects on physical/psychosocial health and cognitive performance in individuals with developmental disabilities.

Besides the behavioral outcomes, neuroimaging assessments, such as structural Magnetic Resonance Imaging (MRI) including Diffusion Tensor Imaging (DTI), functional Magnetic Resonance Imaging (fMRI), Electroencephalogram (EEG), and functional Near-Infrared Spectroscopy (fNIRS), have been used to develop objective measures of abnormalities in brain structure and function associated with the aforementioned developmental disabilities (58). A meta-analytic review of MRI/fMRI studies reported shared as well as distinct structural and functional brain abnormalities in individuals with ASD and ADHD (59). Specifically, gray matter volumes were atypical in the fronto-temporal cortices of individuals with ASD, whereas the orbito-frontal cortices were abnormal for individuals with ADHD (59–61). During cognitive control tasks, atypical prefrontal/precuneus activation was reported in individuals with ASD, whereas fronto-striatal activation abnormalities were reported in individuals with ADHD (59, 62, 63). EEG abnormalities associated with arousal/motivation, inhibitory control, and mental flexibility tasks have also been reported in individuals with ASD, ADHD, and/or LD (64–66). Using fNIRS, atypical fronto-parieto-temporal activation has been reported in infants at risk for and children with ASD during naturalistic, socially embedded actions compared to age-matched controls (67–72). It would be reasonable to expect that physical activity/movement interventions that are known to have cascading effects on psychosocial and cognitive performance may also lead to associated changes in neural activity in the aforementioned neural correlates/biomarkers. Interestingly, while there is some evidence in healthy populations, there is a lack of synthesis of literature for the neural effects of the physical activity/movement interventions in individuals with developmental disabilities. Research studies conducted in healthy subjects have reported associations between neural activity and physical activity levels as well as changes in neural biomarkers post-movement interventions (73–75). Children with higher fitness levels exhibited better inhibitory control and memory, and their structural MRI revealed greater volume in basal ganglia and hippocampus, respectively (76, 77). EEG studies also showed fitness-related differences in functional activity during executive function tasks, with higher fitness levels associated with faster and larger event-related potentials (ERPs, including P3b, N2) and better executive functioning performance (74, 75, 78). Apart from correlational studies, intervention studies have used different neuroimaging tools as objective outcome measures to study the effects of physical activity. A systematic review of endurance-enhancing physical activity interventions found changes in resting-state fMRI and task-related activation in brain regions that are important for attentional control (middle frontal gyrus, superior frontal gyrus, superior parietal lobes, and anterior cingulate cortex) (73). Similarly, a systematic review of resting-state EEG studies also suggested inconsistent, but generally positive training-related changes after exercise interventions, including changes in slow (delta and theta) and fast (alpha and beta) wave activity, indicating normalized cortical-subcortical crosstalk (79). Although more research will need to be conducted, the studies in healthy subjects support the use of neuroimaging tools as objective measures for tracking intervention effects and to understand the neural mechanisms underlying training-related improvements following physical activity/movement interventions.

Compared to healthy populations, fewer studies have investigated the neural mechanisms of physical activity/movement interventions on cognitive and psychosocial functions in individuals with developmental disabilities. To our knowledge, there is no systematic review that provides a broad synthesis of neural effects after physical activity/movement interventions in individuals with developmental disabilities. Therefore, the current systematic review aims to summarize the current neuroimaging findings on chronic and acute effects of physical activity/ movement interventions and quantify effect size estimates for the neural outcome measures. We will assess the utility of neuroimaging tools as objective measures of intervention effects and explain the potential neural mechanisms by which movement interventions promote psychosocial health and cognitive performance in individuals with developmental disabilities.

## Materials and Methods

### Search Strategies

We reviewed literature from four allied health, psychology, physical therapy/kinesiology, and education-related databases, including PubMed (1950–2021), PsycINFO (1969–2021), CINAHL (1937–2021), and Scopus (1966–2021). The search terms included keywords in three areas: (a) Diagnostic terms: Related to neurodevelopmental disorders, including “Autism spectrum disorder,” “Attention-deficit/hyperactivity disorder,” “Developmental coordination disorder”, “Learning disorder,” “Intellectual disorder” …etc.; (b) Intervention terms: Related to motor interventions, including “Sport,” “Exercise,” “Physical activity,” “Intervention,” “Therapy” … etc.; (c) Neuroimaging terms: Related to neuroimaging modalities including “Electroencephalography,” “Magnetic resonance imaging,” “Near infrared spectroscopy,” etc. (detailed search terms in Supplementary Table 1).

### Eligibility Criteria

Studies were included in the review if they fulfilled the following inclusion criteria: (a) Included individuals with developmental disorders (e.g., ASD, ADHD, DCD, LD, ID, developmental delay, etc.), (b) Tested the effects of movement interventions (e.g., physical activity, exercise, yoga, martial arts, etc.), and (c) Used neuroimaging techniques (e.g., fMRI, fNIRS, EEG, etc.) to measure intervention effects. Studies were excluded if the experimental group (a) Only involved sedentary interventions (e.g., applied behavior analysis, speech therapy, education); (b) Were review papers, case reports, and protocol papers; (c) Were in languages other than English; or (d) Were gray literature including theses and dissertations.

There is limited evidence on neural effects of movement interventions, hence, we decided to include individuals across all age ranges (including children, adolescents, adults) and abilities (with or without intellectual disabilities). We included studies on various movement interventions utilizing perceptuomotor skills (i.e., multisystem, creative movement as well as targeted physical activity interventions) with wide-ranging training lengths (i.e., one or more sessions), and that used various structural and functional neuroimaging tools to monitor the training-related neural effects. In terms of study design, we included RCT, controlled clinical trials, and cross-over studies, but not case studies to ensure study quality. Overall, we did not set additional exclusion criteria based on age, ability, or nature/content of the control group interventions.

### Data Extraction and Evaluation

**Figure 1:**
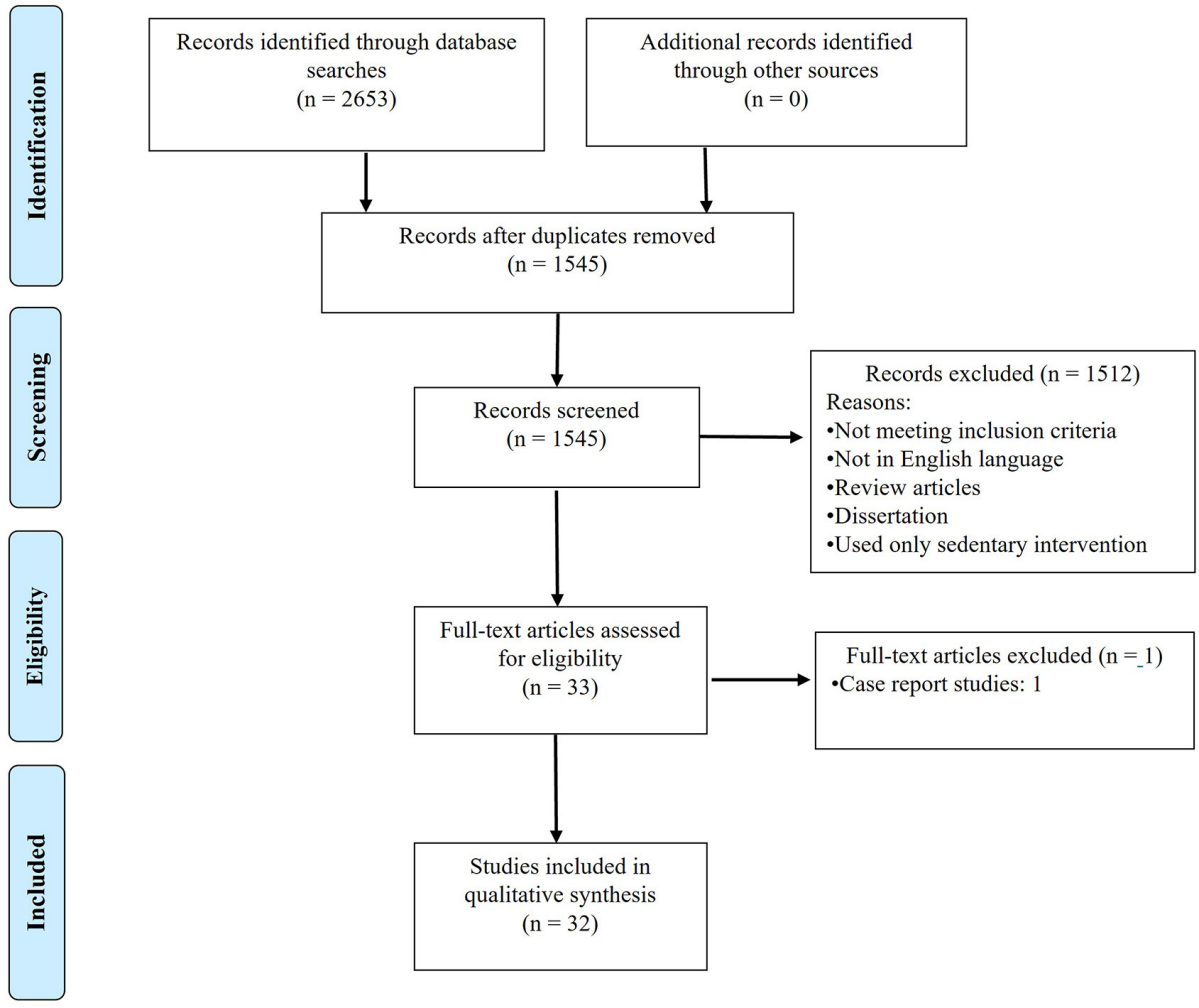
PRISMA diagram for search process.

We conducted our latest database search on Sept 16th, 2021, with a result of 2,653 articles in total (1,036 from PubMed, 453 from PsycINFO, 1,029 from Scopus, and 135 from CINHAL). After removing duplicates and screening through our eligibility criteria, 32 articles qualified for further review (see detailed search process in Figure 1). All authors agreed on the eligibility of 95% of the studies. Disagreements between coders for study inclusion were resolved through consensus meetings.

### Risk of Bias and Level of Evidence Assessments

The current review paper focused on the methodological rigor and quality of study designs used for assessing physical activity/motor intervention effects. Specifically, the Physiotherapy Evidence Database (PEDro) scale was used to assess the risk of bias for RCT, CCT, and cross-over design studies, while the NIH risk of bias assessment (NIH-ROB) was employed for pre-posttest studies with no control group (80, 81). The PEDro scale includes a total of 11 items which are scored on a nominal scale (No = N, Yes = Y) of which 10 items are scored for each study (maximum score 10, the first item on the PEDro is not included in the total score calculations; detailed descriptions in Supplementary Table 2) (80). Note that the PEDro scale criterion #2 requires a study to specifically state that allocation to the intervention was randomized. Allocation procedures using quasi-randomization or counterbalancing did not receive full scores for the criterion of allocation. A PEDro score of more than 6 is classified as high quality, while a score between 4 and 5 is classified as fair, and a score <3 is classified as a low quality study (80). The NIH assessment tool includes 12 items that are also scored on a nominal scale and later summed to give a maximum possible score of 12 (detailed descriptions in Supplementary Table 2) (81). All authors coded 20% of the included articles (6 to 7 articles), and we established inter-and intra-rater reliability of >90%. In addition to the risk of bias assessment tools, we also assessed the levels of evidence of the reviewed papers using the tool designed by Sackett and colleagues (82). Based on the study design, the studies are classified into 5 levels: Level I: RCT or cross-over designs with “high” quality (PEDro Scale score ≥6); Level II: RCT or cross-over designs with “Fair” quality (PEDro Scale score = 4–5) and all CCT designs; Level III: Pre-posttest designs; Level IV: Conflicting evidence of two or more equally designed studies; Level V: RCTs with “Poor quality” (PEDro score ≤3) and case studies or cohort studies/single subject series with no multiple baseline assessments (82).

### Data Extraction and Coding Procedures

For each of the reviewed studies, we extracted information on sample and study characteristics, methodological quality, intervention characteristics (FITT: Frequency, Intensity, Time, Type), neural and behavioral assessments used, dependent variables, and treatment effects using a standard coding template (Supplementary Table 3). Besides narrative descriptions, wherever data was provided in the original reports, we also calculated effect size estimates with their confidence intervals for each outcome measure in reviewed studies to estimate the magnitudes of the treatment effects. Specifically, sample size, means, and standard deviations (and/or standard errors) of the dependent variables were used to calculate the effect sizes using the Hedges' method, a method that is more valid when dealing with smaller sample sizes (*n* < 20) (83). To avoid inaccuracy and allow for fair comparisons between studies, we only calculated the effect sizes if the means and standard deviations (and/or standard errors) for the outcome variables were provided in the reviewed articles.

## Results

### Study Characteristics and Quality Assessments

Of the 32 included articles, 13 were RCTs, 4 were CCTs, 10 were cross-over, and 5 were pre-post test designs (84–115). Seventeen studies examined chronic effects of physical activity/movement intervention, 14 studies examined acute effects only, and 1 study examined both. The PEDro scores of the studies using RCT, CCT, and cross-over designs ranged from 4 to 8 points (Average = 5.56; SD = 1.12), indicating fair to good study quality (Table 1). On the other hand, the NIH risk of bias scores of the studies using pre-post test designs ranged from 6 to 9 points (Average = 7.40; SD = 1.52; Table 2). Because of the nature of movement intervention studies, all CCT, RCT, and cross-over studies did not have the subjects blinded to grouping and the type of intervention they received (PEDro scale item #5, NIH ROB item # 8). Additionally, pre-post test design studies had small samples and a lack of assessment across multiple timepoints before and after interventions (ROB item #s 5 and 11). Although many cross-over design studies used a counterbalancing approach to account for intervention order effects, they did not specify whether their allocation to a certain intervention order was randomized or not, and hence, they did not meet PEDro scale criterion #2 (95, 100–102, 104). In terms of the level of evidence, 12 studies were Level I, 15 were Level II, and 5 were classified as Level III. No included paper was classified as Level IV or V.

**Table 1:** PEDro scores for the CCT, RCT, and cross-over design studies.

**References**	**1**	**2**	**3**	**4**	**5**	**6**	**7**	**8**	**9**	**10**	**11**	**Total**
Bremer et al. (84)	Y	Y	N	Y	N	N	N	Y	Y	Y	Y	6
Cai et al. (85)	Y	N	N	Y	N	N	N	N	Y	Y	Y	4
Chan et al. (86)	Y	Y	Y	Y	N	N	Y	Y	Y	Y	Y	8
Chan et al. (87)	Y	Y	N	Y	N	N	Y	N	Y	Y	Y	6
Corbett et al. (88)	Y	Y	Y	Y	N	N	Y	Y	Y	Y	Y	8
Sharda et al. (89)	Y	Y	N	Y	N	N	Y	Y	Y	Y	Y	7
Yang et al. (90)	Y	Y	Y	Y	N	N	N	N	Y	Y	Y	5
Choi et al. (91)	Y	Y	N	Y	N	N	N	N	Y	Y	Y	5
Chueh et al. (92)	Y	Y	Y	Y	N	N	N	N	Y	Y	Y	6
Huang et al. (93)	Y	N	N	Y	N	N	N	Y	Y	Y	Y	5
Huang et al. (94)	Y	Y	N	Y	N	N	N	Y	Y	Y	Y	6
Hung et al. (95)	Y	N	N	N	N	N	N	Y	Y	Y	Y	4
Janssen et al. (96)	Y	Y	N	Y	N	N	N	N	Y	Y	Y	5
Janssen et al. (97)	Y	Y	N	Y	N	N	N	N	Y	Y	Y	5
Lee et al. (98)	Y	Y	N	Y	N	N	N	N	Y	Y	Y	5
Ludyga et al. (99)	Y	Y	N	Y	N	N	N	Y	Y	Y	Y	6
Mehren et al. (100)	Y	N	N	Y	N	N	N	Y	Y	Y	Y	5
Mehren et al. (101)	Y	N	N	Y	N	N	N	Y	Y	Y	Y	5
Pontifex et al. (102)	Y	N	N	Y	N	N	N	Y	Y	Y	Y	5
Smith et al. (103)	Y	Y	Y	Y	N	N	N	N	Y	Y	Y	6
Tsai et al. (104)	Y	N	Y	Y	N	N	N	N	Y	Y	Y	6
Yu et al. (105)	Y	N	N	Y	N	N	N	N	Y	Y	Y	4
Tsai et al. (106)	Y	N	N	Y	N	Y	Y	Y	Y	Y	Y	7
Tsai et al. (107)	Y	N	N	Y	N	Y	Y	Y	Y	Y	Y	7
Milligan et al. (108)	Y	N	N	Y	N	N	N	Y	Y	Y	Y	5
Chen et al. (109)	N	N	N	Y	N	N	N	Y	Y	N	Y	4
Vogt et al. (110)	Y	Y	N	Y	N	N	N	N	Y	Y	Y	5

*Criteria of the PEDro scale: 1, Eligibility criteria; 2, Group randomization; 3, Concealed allocation; 4, Baseline comparisons; 5, Blinding-subjects; 6, Blinding-therapist; 7, Blinding-assessors; 8, Missing data; 9, Intention to treat; 10, Between-group comparisons; 11, measure of variability (Detailed description in Supplementary Table 1); Y, Yes; N, No; Total scores are calculated by summing the number of met criteria (except the first criterion) for each individual study. A PEDro score ≥ 6 indicates low risk of bias; while a PEDro score < 6 indicates high risk of bias*.

**Table 2:** NIH-ROB scores for the pre-post test studies.

**References**	**1**	**2**	**3**	**4**	**5**	**6**	**7**	**8**	**9**	**10**	**11**	**12**	**Total**
Brand et al. (111)	Y	Y	Y	Y	N	Y	Y	N	Y	Y	N	Y	9
LaGasse et al. (112)	Y	Y	Y	Y	N	Y	Y	N	Y	Y	N	Y	9
Choi et al. (113)	Y	N	Y	N	N	Y	Y	N	Y	N	N	Y	6
Chen et al. (114)	Y	N	Y	N	N	Y	Y	N	Y	Y	N	Y	7
Vogt et al. (115)	Y	N	N	N	N	Y	Y	N	Y	N	N	Y	6

*Criteria of the NIH-ROB scale: 1, Study questions and objective; 2, Eligibility criteria; 3, Representation of general population; 4, Participant enrollment; 5, Sample size; 6, Description of assessment/intervention; 7, Reliability and Validity of the measures; 8, Blinding assessors; 9, Missing data; 10, Pre-postest assessments and p-values; 11, Pre-post-tests in multiple time-points; 12, Statistical analysis including individual-level data (Detailed description in Supplementary Table 1); Y, Yes; N, No; Total scores are calculated by summing the number of met criteria for each individual study*.

### Sample Characteristics

Of the 32 included papers, 16 included individuals with ADHD, 9 included individuals with ASD, 2 included individuals with DCD, 1 included individuals with LD, 3 included individuals with ID, and 1 included individuals with ID and developmental disabilities. The sample size ranged from 4 to 45 per group (Average sample size = 18.01; SD = 7.66). Due to the sex differences in the prevalence of developmental disabilities, most studies included more males than females, with an average male-to-female ratio of about 4.5:1. The majority of the studies included school-age children between 6 and 18 years (25 of the 32 studies), 2 studies included preschoolers (3–5 years), and 5 included adults with developmental disabilities (>18 years). Twenty out of the 32 studies reported the mean IQ of their participants (76.3–121.3 across studies) with only 6 studies including children with ID (Tables 3–**6**).

**Table 3:** Study characteristics of studies assessing the chronic effects of physical activity/movement interventions (ADHD, LD, ID).

**References**	**Design/evidence level**	**Sample (E/C)**	**Age (E/C: M ± SD; Range)**	**Gender (E/C)**	**IQ (E/C)**	**Movement int**	**Min/s; s/wk; # of WKs**	**Intensity (% HR max/mean ±SD)**	**Control int**	**Neural measure**	**Task**
**Attention-Deficit/hyperactivity disorder**											
Cho et al. (91)	RCT/II	13/17	15.8 ± 1.7/ 16.0 ± 1.2; 13–18	13M0F/17M0F	94.9 ± 11.8/ 95.9 ± 15.2	Aerobic exercise	90; 6; 18	60%	Behavioral intervention	fMRI	Mental flexibility
Huang et al. (93)	CCT/II	15/14	7.9 ± 1.0/ 8.3 ± 1.0; 5–10	11M4F/14M0F	–/–	Water aerobic	90; 2; 8	50–60%	–	EEG	Resting state
Janssen et al. (96)	RCT/II	24/25	9.8 ± 2.0/ 9.2 ± 1.3; –	19M5F/19M6F	98.3 ± 13.8/ 100.8 ± 14.3	Physical activity	35;–;– (28 s)	70–100%	Medication	EEG	Inhibitory control
Janssen et al. (97)	RCT/II	27/25	9.8 ± 1.9/ 9.1 ± 1.1; –	21M6F/19M6F	>80/>80	Physical activity	35;–;– (28 s)	70–100%	Medication	EEG	Inhibitory control
Lee et al. (98)	RCT/II	6/6	8.8 ± 1.0/ 8.8 ± 1.0; –	6M0F/6M0F	>80/>80	Combined exercise	60; 3; 12	45–75%	–	EEG	Resting state & mental flexibility
Smith et al. (103)	RCT/I	13/16	7.2 ± 1.4/ 7.1 ± 1.1; 5–9	7M6F/8M8F	107.5 ± 14.7/ 99.3 ± 11.2	Integrated brain, body, and social	120; 3; 15	–	–	EEG	Inhibitory control
**Learning disabilities**											
Milligan et al. (108)	CCT/II	45/36	13.1 ± 1.7/ 12.8 ± 1.2; 11–17	41M7F/31M7F	–/–	Martial art	90; 1; 20	–	–	EEG	Inhibitory control & auditory attention
**Intellectual disabilities**											
Chen et al. (109)	CCT/II	14/4	22.4 ± 1.9/ 22.01 ± 1.8; –	10M4F/4M0F	–/–	Badminton	50; 5; 10	–	–	EEG	Resting state

*RCT, Randomized control trials; CCT, Controlled clinical trial; Pre-Post, Pre-post-test design; E, Experimental group; C, Comparison group; M, Mean; SD, Standard deviation; M, Male; F, Female; IQ, Intelligence quotient; INT, Intervention; MIN, Minute; S, Session; WK, Week; HR MAX, Maximum heart rate; ABA, Applied Behavior Analysis; SENSE, Social Emotional NeuroScience Endocrinology; EEG, Electroencephalogram; DTI, Diffusion tensor imaging; fMRI, Functional magnetic resonance imaging*.

### Frequency, Intensity, Time, and Type of Movement Interventions

Of the 32 included papers, 17 assessed the chronic effects of multiple intervention sessions, 14 assessed the acute effects after a single bout of exercise, and 1 assessed both the acute and the chronic effects of movement interventions. In the following paragraphs, we describe the Frequency, Intensity, Time, and Type (FITT) of interventions provided in the included studies (Tables 3–**6**).

#### Frequency

For the studies that assessed the chronic effect of exercise, the intervention frequency ranged from 1 to 5 sessions per week (average: 2.69; SD: 1.35), and intervention duration ranged between 3 and 20 weeks in total (average: 9.50; SD: 4.90). Therefore, the total training volume ranged from 9 to 60 sessions (average: 26.30; SD: 18.78; Tables 3, 4). For the studies that assessed the acute effects of movement intervention, only one 1 session was conducted (Tables 5, 6).

**Table 4:** Study characteristics of studies assessing the chronic effects of physical activity/movement interventions (ASD and DCD).

**References**	**Design/evidence level**	**Sample (E/C)**	**Age (E/C: M ±SD; Range)**	**Gender (E/C)**	**IQ (E/C)**	**Movement int**	**Min/s; s/Wk; # of WKs**	**Intensity (% HR max/ mean±SD)**	**Control int**	**Neural measure**	**Task**
**Autism spectrum disorder**											
Brand et al. (111)	Pre-post/III	10/–	10.0 ± 2.3/–; 7–13	5M5F/–	–/–	Cycling & motor skill training	60; 3; 3	–/–	–	EEG	Sleep
Cai et al. (85)	CCT/II	15/14	5.1 ± 0.6/ 4.6 ± 0.7; 3–6	2M3F/13M1F	–/–	Mini-Basketball training	40; 5; 12	–/ 136.0 ± 6.1	ABA training	DTI	Resting state
Chan et al. (86)	RCT/I	20/20	11.3 ± 3.9/ 12.4 ± 3.3; 6–17	19M1F/17M3F	78.4 ± 18.9/ 80.5 ± 18.5	Nei Yang Gong	60; 2; 4	–/–	Muscle relaxation	EEG	Inhibitory control
Chan et al. (87)	RCT/I	18/17	11.9 ± 4.1/ 11.0 ± 3.3; 5–17	17M1F/15M2F	76.3 ± 17.7/ 86.5 ± 17.5	Nei Gong	60; 2; 4	–/–	Muscle relaxation	EEG	Visual memory
Corbett et al. (88)	RCT/I	17/13	11.38 ± 2.5/ 10.7 ± 1.9; 8–14	13M4F/11M2F	100.1 ± 16.8/ 95.9 ± 21.2	SENSE-theater treatment	240; 1; 10	–/–	Waitlist	EEG	Face memory
LaGasse et al. (112)	Pre-post/III	7/–	8.4 ± 2.9/–; 5–12	6M1F/–	–/–	Music therapy	35; 2; 5	–/–	–	EEG	Sensory gating
Sharda et al. (89)	RCT/I	26/25	10.3 ± 1.9/ 10.2 ± 1.9; 6–12	21M5F/22M3F	102.0 ±18.8/ 94.0 ± 18.2	Music therapy	45; 1; 8 ~ 12	–/–	Play-Based intervention	fMRI	Resting state
Yang et al. (90)	RCT/II	15/15	4.7 ± 0.7/ 5.0 ± 0.6/ 3–6	13M2F/12M3F	–	Mini-Basketball training	40; 5; 12	60–69%/–	Behavioral intervention	fMRI	Resting state
**Developmental coordination disorder**											
Tsai et al. (104)	RCT/I	16/14	9.7 ± 0.4/ 9.5 ± 0.3; 9–10	9M7F/9M5F	104.6 ± 5.7/ 103.4 ± 6.1	Soccer training	50; 5; 10	–	–	EEG	Inhibitory control
Tsai et al. (107)	RCT/I	20/20	11.5 ± 0.3/ 11.5 ± 0.3; 11–12	13M7F/12M8F	108.0 ± 6.5/ 108.4 ± 7.1	Aerobic exercise	50; 3; 16	80–90%	–	EEG	Working memory

*RCT, Randomized control trials; CCT, Controlled clinical trial; Pre-Post, Pre-post-test design; E, Experimental group; C, Comparison group; M, Mean; SD, Standard deviation; M, Male; F, Female; IQ, Intelligence quotient; INT, Intervention; MIN, Minute; S, Session; WK, Week; HR MAX, Maximum heart rate; ABA, Applied behavior analysis; SENSE, Social emotional NeuroScience endocrinology; EEG, Electroencephalogram; DTI, Diffusion tensor imaging; fMRI, Functional magnetic resonance imaging*.

**Table 5:** Study characteristics of studies assessing the acute effects of physical activity/movement interventions (ADHD).

**References**	**Design/evidence level**	**Sample (E/C)**	**Age (E/C: M ±SD; Range)**	**Gender (E/C)**	**IQ (E/C)**	**Movement int**	**Time (min)**	**Intensity (% HR max)**	**Control int**	**Neural measure**	**task**
**Attention-Deficit/hyperactivity disorder**											
Choi et al. (113)	Pre-post/III	27/–	–/–; 12–14	14M13F/–	91–113/–	Dynamic stretching exercise	13	–/–	–	EEG	Resting state
Chueh et al. (92)	RCT/I	E1: 14 E2: 15/ C: 17	E1: 10.1 E2: 9.6/ C:10.4; 7–12	E1: 14M0F E2: 15M0F/C: 16M1F	**–/–**	Treadmill running	E1: 50; E2: 30	50–70%	Video watching	EEG	Resting state
Huang et al. (94)	Cross-Over/I	24/24	9.5 ± 1.6/ 9.5 ± 1.6; 7–12	24M0F/24M0F	105.7 ± 9.0/ 105.7 ± 9.0	Treadmill running	30	65–75%	Video watching	EEG	Resting state
Hung et al. (95)	Cross-Over/II	34/34	10.2 ± 1.7/ 10.2 ± 1.7; 8–12	33M1F/33M1F	104.9 ± 16.9/ 104.9 ± 16.9	Treadmill running	30	50–70%	Video watching	EEG	Mental flexibility
Ludyga et al. (99)	Cross-Over/I	E1: 14 E2: 14/ C: 14	E1: 12.8 ± 1.8; E2: 12.8 ± 1.8/ C: 12.8 ± 1.8	E1: 11M5F; E2: 11M5F/ C:11M5F	–/–	E1: Coordination E2: Cycling	E1: 20 E2: 20	E1:–; E2: 65–70%	Video watching	EEG	Inhibitory control
Mehren et al. (100)	Cross-Over/II	20/20	29.9 ± 9.5/ 29.9 ± 9.5; –	16M4F/16M4F	–/–	Cycling	30	50–70%	Video watching	fMRI	Inhibitory control & visual attention
Mehren et al. (101)	Cross-Over/II	20/20	31.4 ± 9.6/ 31.4 ± 9.6; –	17M3F/17M3F	–/–	Cycling	30	50–70%	Video watching	fMRI	Inhibitory control
Pontifex et al. (102)	Cross-Over/II	20/20	–/–; 8–10	14M6F/14M6F	110–121/110–121	Treadmill running	20	65–75%	Seated reading	EEG	Inhibitory control
Tsai et al. (104)	Cross-Over/I	25/25	10.5 ± 1.2/ 10.5 ± 1.2; –	23M2F/23M2F	–/–	Treadmill running	30	E1: 30%; E2: 50–60% E3: 70–80%	–	EEG	Resting state & inhibitory control
Yu et al. (105)	Cross-Over/II	24/24	9.9 ± 1.3/ 9.9 ± 1.3; 8–12	23M1F/23M1F	105.0 ± 9.8	Treadmill running	30	60–70%	Video watching	EEG	Inhibitory control

*Pre-Post, Pre-post-test design; Cross-over, Cross-over design (note that the same group of participants act as the experimental and comparison groups); E, Experimental group (E1, the first experimental group; E2, the second experimental group; E3, the third experimental group); C, Comparison group; M, Mean; SD, Standard deviation; M, Male; F, Female; IQ, Intelligence quotient; INT, Intervention; MIN, Minute; HR MAX, Maximum heart rate; EEG, Electroencephalogram; fNIRS, functional near infrared spectroscopy; fMRI, Functional magnetic resonance imaging*.

**Table 6:** Study characteristics of studies assessing the acute effects of physical activity/movement interventions (ASD & ID).

**References**	**Design/evidence level**	**Sample (E/C)**	**Age (E/C: M ±SD; Range)**	**Gender (E/C)**	**IQ (E/C)**	**Movement int**	**Time (min)**	**Intensity (% HR max/mean±SD)**	**Control int**	**NeurAL measure**	**Task**
**Autism spectrum disorder**											
Brand et al. (111)	Pre-post/III	10/–	10.0 ± 2.3/–; 7–13	5M5F/–	–/–	Aerobic bicycle & motor skill training	60	–/–	–	EEG	Sleep
Bremer et al. (84)	Cross-Over/I	12/12	11.1 ± 1.3/ 11.1 ± 1.3; –	12M0F/12M0F	–/–	E1: Circuit; E2: Treadmill	20	60–80%/–	Video watching	fNIRS	Inhibitory control & Sustained attention
**Intellectual disabilities**											
Chen et al. (114)	Pre-post/III	12/12	DS: 21.3 ± 5.4; ASD: 18.5 ± 2.0 FXS: 26.17	8M4F/–	–	Treadmill running	20	<85%	–	EEG	Resting state
Vogt et al. (115)	Pre-post/III	11/11	22.5 ± 9.9/–; –	12M0F/–	–	Running	30	–; 154.5 ± 14.4	–	EEG	Resting state
Vog et al. (110)	Cross-Over/II	11/11	16.0 ± 1.34/ 16.0 ± 1.34; –	6M5F/6M5F	–	Cycling	10	–; 143.1 ± 14.4	Music listening	EEG	Resting state & decision making

*Pre-Post, Pre-post-test design; Cross-over, Cross-over design (note that the same group of participants act as the experimental and comparison groups); E, Experimental group (E1, the first experimental group; E2, the second experimental group; E3, the third experimental group); C, Comparison group; DS, Down syndrome; ASD, Autism spectrum disorder; FXS, Fragile X syndrome; M, Mean; SD, Standard deviation; M, Male; F, Female; IQ, Intelligence quotient; INT, Intervention; MIN, Minute; HR MAX, Maximum heart rate; EEG, Electroencephalogram; fNIRS, functional near infrared spectroscopy; fMRI, Functional magnetic resonance imaging*.

#### Intensity

Most studies reported training intensity using target heart rate which was expressed as a percentage of the maximum heart rate of the individual. Of the studies that assessed the chronic effects of the interventions, 7 reported the target heart rate of their movement interventions ranging from 45 to 100% of the suggested maximum heart rate (Tables 3, 4). Specifically, 1 study used intervention with light to moderate intensity (<70% maximum heart rate), 3 studies used moderate intensity activities (50–70% maximum heart rate), and 3 studies provided moderate to vigorous intensity activities (>50% maximum heart rate). One study additionally reported an average heart rate of 135.97 bpm (85). For the studies that assessed the acute effects of interventions, 7 reported the target heart rate of their movement intervention, ranging from 50 to 80% of the suggested maximum heart rate (moderate to vigorous intensity activity; Tables 5, 6). One study included three experimental groups with the target exercise heart rate of 30% (Light), 50–60% (Moderate), and 70–80% (Vigorous) of the suggested maximum heart rate, respectively (104). Two other studies reported average heart rate during/right after movement intervention (Vogt et al. (115): 154.50 bpm ± 10.06; Vogt et al. (110): 143.09 bpm ± 14.40; Table 6).

#### Time

The training time for the studies that assessed the chronic effects of exercise varied widely from 35 to 240 min, with an average of session time 69.44 min (SD = 48.69). The Social Emotional NeuroScience Endocrinology (SENSE-theater) treatment had the longest training time (240 min/session) (88), while the music therapy and physical activity interventions had the shortest training time (35 min/session; Tables 3, 4) (96, 97, 112). For studies that assessed the acute effects of exercise, the training time ranged from 10 to 60 min, with an average training time of 27.53 min/session (SD = 11.33; Tables 5, 6).

#### Type

For the studies that assessed chronic effects, 6 involved sustained aerobic exercises (i.e., running, cycling, stepping, jump rope activities), 2 involved circuit-based exercises with short resting bouts, 7 involved ball-related exercises (i.e., throw and catch, basketball, soccer, badminton), 3 involved martial art training (i.e., Nei-Yang Gong), 3 specifically targeted motor skills (including balance, coordination, and strength), 2 involved the use of musical instruments, 1 involved dancing, 1 involved cognitive games, and 1 used theatrical settings (Tables 3, 4). The majority of the studies (12 of 14) that assessed acute effects of exercise used aerobic exercises including cycling and treadmill walking/running (Tables 5, 6). In addition, 1 study used circuit training with short resting bouts, 2 targeted motor skills (i.e., balance and coordination), and 1 specifically focused on dynamic stretching exercises.

### Comparison Group Interventions

For the studies that assessed chronic effects, 9 did not provide intervention to the comparison group, 1 provided applied behavior analysis training, 2 used muscle relaxation techniques, 1 used a play-based intervention, 2 provided behavioral education, 2 provided medications, and 1 used a waitlist control design (Tables 3, 4). For studies that assessed acute effects, 1 did not include a comparison group, 4 did not provide intervention to the comparison group, 8 asked participants to watch a video, 1 had them listen to music, and 1 had the children involved in seated reading activities (Tables 5, 6).

### Neuroimaging Assessments

The majority of the studies (25 out of 32) used EEG to assess the neural effects of movement interventions. Other neuroimaging tools that were used included functional Magnetic Resonance Imaging (fMRI; *n* = 5), functional Near-Infrared Spectroscopy (fNIRS; *n* = 1), and Diffusion Tensor Imaging (DTI; *n* = 1). Among the 23 EEG studies, one study used sleep EEG to determine the sleep quality in participating children (111), while the remaining studies recorded neural activity during resting-state (*n* = 6), or during different functional tasks assessing inhibitory control (*n* = 9), mental flexibility (*n* = 3), memory (*n* = 3), sensory gating (*n* = 1), and auditory attention (*n* = 1). The EEG variables included: (1) Slow waves (Theta band), fast waves (Alpha and Beta bands), and their ratios (i.e., Theta/Alpha and Theta/Beta ratios) (*n* = 8). Note that the slow and fast wave activity is associated with cortical arousal, (2) Amplitude and latency of event-related potentials (ERP) during executive functioning tasks (i.e., positive peaks in the ERP waveform such as P3b and negative peaks in ERP waveform such as N2) (*n* = 11). Note that greater P3b/N2 amplitude and shorter latency are indicative of more efficient cognitive processing during inhibitory control tasks, (3) Level of right-left frontal asymmetry (*n* = 2), with increased asymmetry indicating greater motivation during exercise, and (4) Sleep EEG variables (i.e., total sleep time, duration of rapid eye movements, etc.) (*n* = 1).

The 5 fMRI studies reported Blood-Oxygen-Level-Dependent (BOLD) signals and functional connectivity (i.e., associations or activity) during resting-state (*n* = 2), or during tasks assessing inhibitory control (*n* = 1), mental flexibility (*n* = 1), or both inhibitory control and attention (*n* = 1). Greater levels of the BOLD signal indicate greater brain activation, while greater connectivity indicates increased synchronized neural activity between brain regions. Only one DTI study measured fractional anisotropy (FA) and mean diffusivity (MD) in brain tissues during sedation/resting state (85). The FA and MD measures are indicative of white matter fiber density, axonal diameter, and myelination, with increased FA and decreased MD reflecting altered white matter organization. Lastly, one fNIRS study recorded the concentration of oxyhemoglobin during an inhibitory control task (84); Typically, higher levels of oxyhemoglobin (Oxy-Hb) indicate greater activation in measured brain regions (details in Tables 3–6).

### Chronic and Acute Neural Effects of Movement Interventions

**Figure 2:**
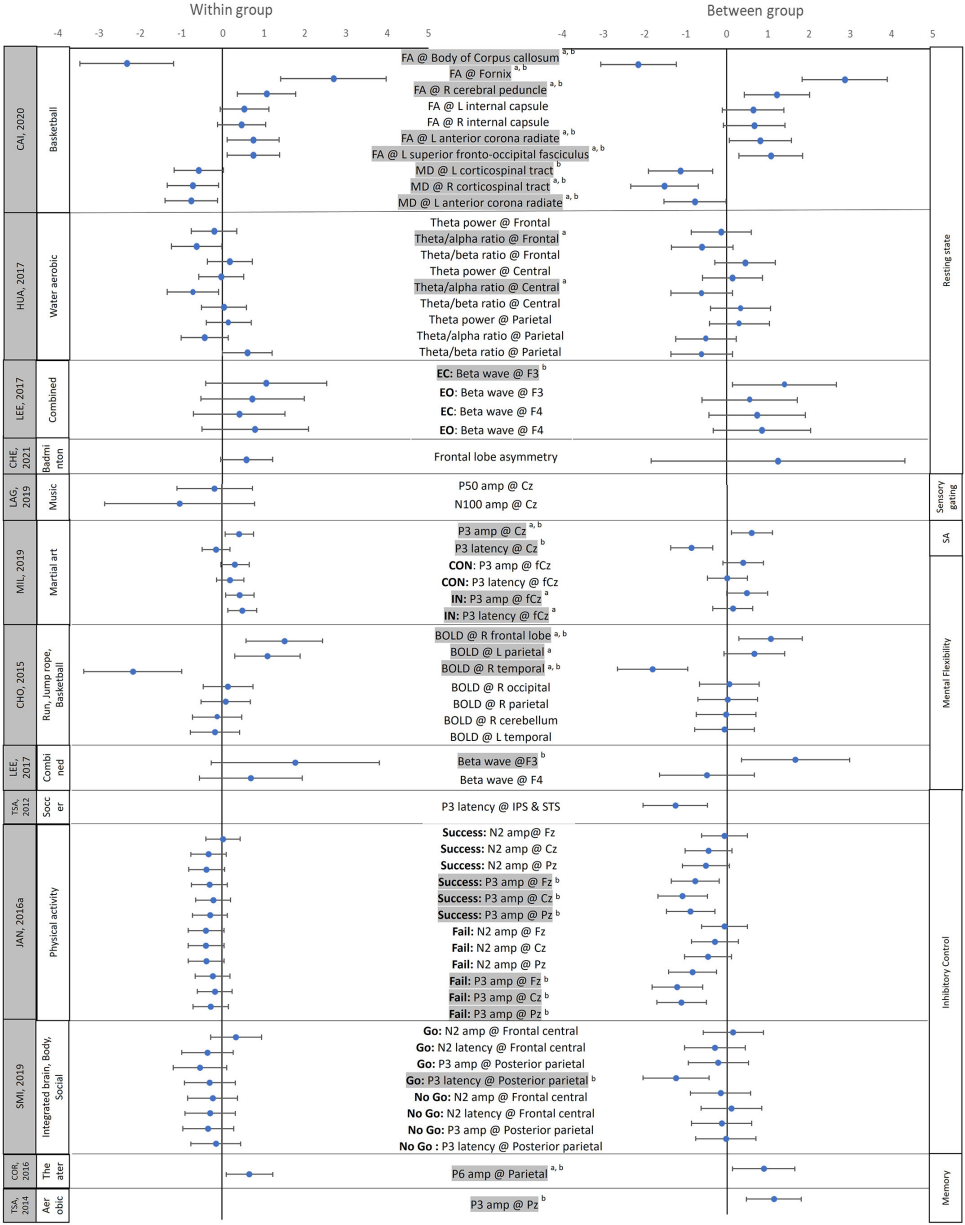
Effect sizes for the chronic neural effects after movement interventions. The mean (solid circle) and 95% CI of the Hedges' g effect sizes were provided for studies assessing the chronic effects of physical activity/movement interventions. The data on the left side shows the effect sizes for within-group comparisons (pre vs. post), while the data on the right side shows effect sizes for between-group comparisons (Experimental group vs. control group); ^a^Shows that the 95% CIs of within-group comparisons does not include 0; ^b^Shows that the 95% CIs of between-groups comparisons does not include 0. Shaded variable indicates that the 95% CIs of between and/or the within-group comparisons does not include 0. FA, Fractional anisotropy; MD, Mean diffusivity; L, left; R, right; EC, Eyes closed; EO, Eyes opened; Go, the go condition during the Go-no-go task; No go, the no go condition during the Go-no-go task; CON, Congruent condition during the Flanker task, IN, incongruent condition during the Flanker task; Success, the trials when the participants successfully inhibited the impulses; Fail, the trials when the participants failed to inhibit the impulses; SA, selective attention; Fz, Cz, Pz, FCz, CPz refer to the locations on the head according to the international 10–20 system; amp, amplitude. Please note that to ensure accuracy and to allow between-study comparisons, this table only includes the effect sizes of the outcome variables for which the means, standard deviation/standard error of means, and study sample sizes were provided by the original papers.

**Figure 3:**
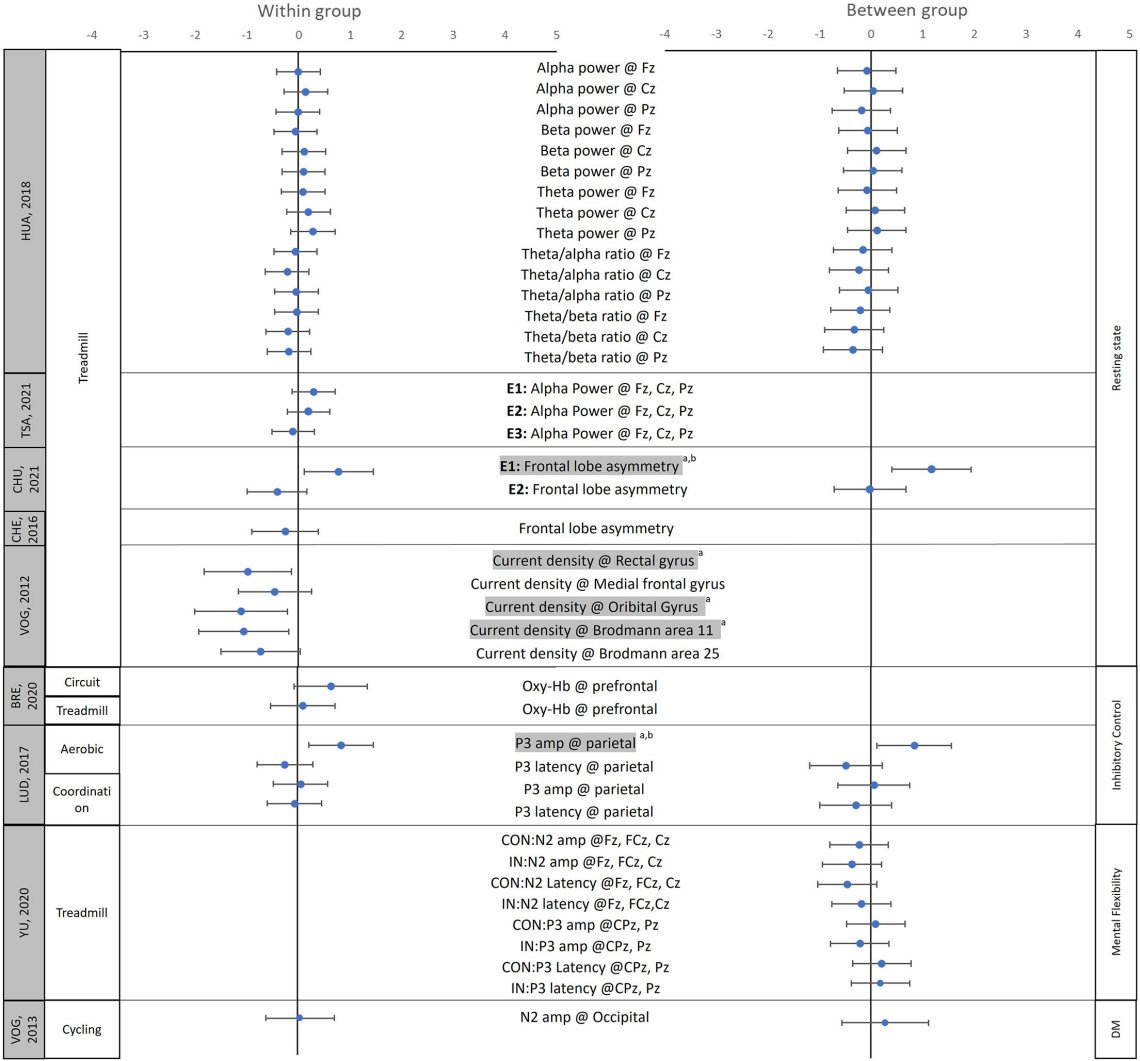
Effect sizes of the acute neural effects after movement interventions. The mean (solid circle) and 95% CI of the Hedges' g effect sizes were provided for the studies focused on the acute effects of the physical activity/movement interventions. The data on the left side show the effect sizes for within-group comparisons (pre vs. post), while the data on the right side show the effect sizes for between-group comparisons (Experimental group vs. control group); ^a^Shows that the 95% CIs of within-group comparisons does not include 0; ^b^Shows that the 95% CIs of between-groups comparison does not include 0. Shaded variable indicates that 95% CIs of between- and/or within-group comparisons does not include 0; L, left; R, right; CON, Congruent condition during the Flanker task, IN, incongruent condition during the Flanker task; DM, decision making; Fz, Cz, Pz, FCz, CPz refer to the locations on the head according to the international 10–20 system. Oxy-Hb, concentration of the oxygenated hemoglobin; amp, amplitude. Please note that to ensure accuracy and to allow between-study comparisons, this table only includes the effect sizes of the outcome variables for which the means, standard deviation/standard error of means, and study sample sizes were provided by the original papers.

Sixteen of the 18 studies that assessed chronic effects of movement interventions reported positive effects on at least one neural measure, whereas 12 of the 15 studies that assessed the acute effects of exercise reported significant beneficial effects in at least one neural measure after a single bout of exercise (Supplementary Tables 4, 5). We were able to calculate effect sizes for 13 chronic and 9 acute effect studies based on the means and standard deviations (and/or standard errors) provided in the publications. The effect sizes of the chronic effect studies ranged from −2.34 to 2.87 (negative effect sizes indicate reduced neural activity post-intervention), with 10 studies having the 95% confidence intervals (CI) of at least 1 variable not including 0 (Figure 2; Supplementary Table 6). The effect sizes of the acute effect studies ranged from −1.1 to 1.17, with 3 studies having the 95% CI of at least 1 variable not including 0 (Figure 3; Supplementary Table 6). Although more studies are needed to investigate the differences between chronic and acute effects of movement intervention, the current literature confirms larger effect sizes following multiple sessions (chronic) vs. a single training session (acute).

### Structural and Functional Changes as Well as Domain-Specific Neural Effects of Movement Interventions

#### Structural Organization

Using DTI, one study investigated the training-related changes in the structural organization of brain tissue (85). Specifically, Cai et al. found training-related improvements in social responsiveness and normalized fractional anisotropy in the fornix, fronto-occipital fasciculus, cerebellar peduncle, internal capsule, anterior corona radiate [Hedges' g = 0.46–2.70 (within); −1.51 to 2.87 (between)], as well as decreased mean diffusivity in bilateral corticospinal tracts [Hedges' g = −0.58 and −0.72 (within); −1.12 to −1.51 (between)] after 12 weeks of mini-basketball training in children with ASD (85).

#### Sleep Quality

There was one study that investigated the chronic and acute effects of a movement intervention on sleep quality in children with ASD (111). Specifically, Brand et al. conducted sleep EEG in children with ASD before and after 3 weeks of aerobic exercise and motor skill intervention (chronic effects) and assessed acute effects of the intervention by collecting EEG data during nights preceding the intervention as well as nights preceding days when no intervention was provided (acute effects) (111). They found improved sleep quality in children with ASD (higher sleep efficiency, % of deep sleep, % slow-wave sleep, and reduced sleep onset latency) in the nights preceding intervention days compared to the nights preceding days without intervention (positive acute effect; absolute Hedges' g = 0.15–1.39 (within); Supplementary Table 6) (111). Although there was no significant chronic effect of the movement intervention on sleep quality as assessed using the Sleep EEG measure [absolute Hedges' g = 0.04–0.75 (within); Supplementary Table 6], better ball skills and balance performance were reported after 3 weeks of aerobic and motor skill intervention (See Supplementary Tables 4, 5). In short, there were greater acute compared to chronic effects of physical activity on sleep quality in children with ASD.

#### Emotional Responses to Movement Interventions

Three studies investigated the changes in EEG resting-state frontal asymmetry after chronic and acute movement interventions in children with ID and ADHD (92, 109, 114). Typically, greater left than right frontal activity is associated with motivation to continue physical activity/tasks, whereas greater right than left frontal activity is associated with lower levels of motivation to pursue physical activity/tasks. Although Chen et al. found reduced left frontal asymmetry after 20 min of treadmill running exercise, indicating low motivation to adhere to exercises [Hedges' g = −0.26 (within)] (114); Chueh et al. found increased left frontal asymmetry after 50 min of treadmill running compared to 30 min of treadmill running and sedentary video watching [E1 (50 min): Hedges' g = 0.78 (within), 1.17 (between); E2 30 min: Hedge' g = −0.41 (within), −0.02 (between)] (92). Moreover, Chen et al. found increased left frontal asymmetry after 10 weeks of badminton training, indicating better motivation to engage in a chronic ball skill intervention [Hedges' g = 0.59 (within), −0.66 (between); Supplementary Tables 4–6] (109). Due to the inconsistent results, more studies are needed to understand how duration and types of physical activity/ movement intervention might lead to different levels of motivation to pursue exercise and the subsequent effects on exercise adherence (indicated by left frontal asymmetry).

#### Resting-State Cortical Arousal

Using EEG, several studies found changes in resting-state slow- and fast-wave activity in children with ADHD, suggesting normalized cortical arousal level after movement interventions (91, 93, 94, 97, 104). Specifically, Janssen et al. (2016) found decreased theta activity over the midline regions (Fz, Cz, and Pz) after 28 physical activity training sessions (97), and Huang et al. found decreased EEG theta/alpha ratios over frontal (F2, F4, Fz) and central (C3, C4, Cz) regions following an 8-week water aerobics intervention compared to a control intervention [Hedges' g = −0.63 and −0.72 (within); −0.60 and −0.61 (between)] (93). Similar results were found in acute effect studies, with Huang et al. reporting reduced theta/beta ratios in the midline regions [Hedges' g = −0.04 to −0.20 (within); −0.21 to −0.35 (between)] (94), Tsai et al. reporting increased alpha power after a single bout of treadmill running [E1: Hedges' g = 0.29; E2: Hedges' g = 0.20; E3: Hedges' g = −0.11 (within)] (104), and Choi et al. (113) reporting increased alpha band and reduced theta band/theta-beta ratio after a single bout of dynamic stretching exercise, indicating improved normalized cortical arousal (113). Although the associations between neural and behavioral/symptoms remain to be explored, our review of the literature suggests that both chronic and acute movement interventions seem to lead to normalized cortico-subcortical crosstalk in children with ADHD (Supplementary Tables 4–6).

#### Resting State Connectivity

fMRI studies found training-related changes in resting-state neural activity in regions important for social communication skills in children with ASD (89, 90). Yang et al. (90) found increased connectivity between the left inferior frontal gyrus and the right cerebellum after mini-basketball training (90). Similarly, Sharda et al. found reduced resting-state fMRI over-connectivity between the auditory and visual regions and under-connectivity between the auditory and motor regions after 8–12 weeks of music therapy in children with ASD (89). Moreover, the changes in connectivity were associated with improvements in communication skills in children (89). Overall, these findings suggest that movement interventions might benefit the social communication performance of children with ASD through more efficient social/motor information transmission (Supplementary Tables 4–6) (89, 90).

#### Inhibitory Control

Using different inhibitory control tasks (including Stop sign task, Go-no-Go, Flanker tasks, Visuospatial attention paradigm, decision making, and the attention sustained subtest of the Leiter international performance scale), multiple studies found improved behavioral performance and/or associated neuroplastic changes in EEG/fNIRS/fMRI neural activity after movement interventions in individuals with ADHD, ASD, DCD, and LD (84, 86, 96, 97, 99–103, 105, 106, 108, 110). In terms of behavioral effects of movement interventions, although a few studies failed to report significant changes in inhibitory control performance in children with developmental disabilities (101, 103, 108), others reported increased response accuracy (84, 102, 105), and reduced reaction time during inhibitory control tasks (99, 100, 106, 110), as well as improved parent-reported performance in tasks assessing self-control abilities in individuals with developmental disabilities (86).

For the EEG-related neural effects, the P3b and N2 amplitude/latency were two of the most frequently studied ERP components during inhibitory control tasks (96, 99, 102, 103, 105, 108). Overall, movement interventions led to a normalization of EEG neural activity, including increased amplitude of P3b [Hedges' g = 0.31–0.84 (within), 0.39–0.84 (between)] (99, 102, 108), and N2 peak [Hedges' g = −0.39 to 0.02 (within), −0.06 to −0.52 (between)] (96, 105), as well as reduced latency of P3b [Hedges' g = −0.15 to −0.30 (within), −0.03 to −1.26 (between)] (102, 103, 106), and N2 waves [Hedges' g = −0.18 to −0.45 (between)] (105, 110). Similarly, using fNIRS, Bremer et al. (2020) found increased oxyhemoglobin concentration over the prefrontal cortex following circuit training but not after a treadmill training intervention [Hedges' g = 0.64 and 0.10 (within), respectively] (84). For fMRI-related neural effects, two papers from the same research group found increased activation over the temporal (superior and middle temporal regions), parietal (i.e., superior and inferior parietal gyri, postcentral and supramarginal gyri), and occipital lobes during Go-no-go tasks (100), but no significant changes in brain activity during the Flanker task after a single bout of cycling exercise (101). Taken together, despite some inconsistent results, several studies found improved inhibitory control along with normalized EEG and higher levels of activation in task-appropriate neural substrates using fMRI/fNIRS (Supplementary Tables 4–6).

#### Mental Flexibility

Three studies investigated the effects of the movement-related intervention on mental flexibility in children with ADHD and found improved behavioral performance (91, 95, 98). Specifically, Lee et al. found increased color-word score during the Stroop task after 12 weeks of combined exercise (98); Hung et al. found improved reaction times during task switching after 30 min of treadmill running (95); Choi et al. found fewer preservation errors during Wisconsin Card Sorting test after the 13 min of dynamic stretching exercise (91). For neural activity findings, Lee et al. found increased EEG beta wave activity over the frontal regions (F3 & F4) in children with ADHD after 12 weeks of combined movement exercise (including balancing, jumping rope, and stretching) [Hedge' g = 0.70–1.77 (within), −0.49–1.66 (between)] (98). Similarly, Hung et al. (2016) found increased P3b amplitude over the midline regions (Fz, Cz, Pz) during rule-shifting than non-shifting conditions after a single bout of treadmill running (95). Lastly, using fMRI, Choi et al. (2015) found increased activation over the right frontal and left parietal regions [Hedges' g = 1.10–1.51 (within), 0.66–1.05 (between)], as well as decreased activation over the temporal lobe after 18 weeks of aerobic exercises [Hedges' g = −2.17 (within), −1.81 (between)] (91). Taken together, both acute and chronic movement-related interventions have positive effects on mental flexibility and led to normalized EEG and fMRI neural activity important for selective attention and stimulus processing/discrimination (Supplementary Tables 4–6) (91, 95, 98).

#### Memory

Two studies that focused on visual memory performance in children with ASD and one study that focused on the visuospatial working memory in children with DCD found positive effects of the movement-related intervention on behavioral memory tests as well as underlying neural activity performance along with training-related changes in neural activity (87, 88, 107). Specifically, while Chan et al. found enhanced memory (increased total recall) and better memory retrieval strategies (increased semantic clustering and visual scanning performance) in children with ASD after 4 weeks for Nei Yang Gong/martial art training (87), Corbett et al. found improved memory of faces with and without a delayed period in children with ASD after 10 weeks of SENSE-theater intervention (88). Similarly, Tsai et al. found enhanced response accuracy during a visuospatial working memory task (i.e., remember the spatial locations of ladybirds) in children with DCD after 16 weeks of aerobic exercise (107).

For neural effects, Chan et al. (2015) found a training-related increase in EEG theta coherence over the frontoposterior regions, indicating better cortical connectivity between brain regions (87). On the other hand, Corbett et al. (2016) found normalized ERP amplitude between 300 and 500 ms after stimuli over the parietal lobe, after SENSE theater intervention, indicating enhanced working memory (88). Lastly, Tsai et al. found increased P3b amplitude over the frontal, central, temporal, parietal, and occipital regions during the retrieval process when working on the visuomotor working memory task [Hedges' g = 1.13 (between-group)] (107). Movement-related interventions might have positive effects on memory performance including visuospatial memory, memory of faces, and working memory, and lead to changes in neural activity important for resource allocation during the retrieval process (Supplementary Tables 4–6) (87, 88, 107).

### Associations Between Neural and Behavioral Improvements

Few studies reported the correlation between neural and behavioral improvements after movement-related intervention (91, 96, 105). Using EEG, Janssen et al. found a significant but relatively weak positive association between changes in N2 amplitude over Cz and improvements in inhibitory control (indicated by the change of reaction time during Stop Sign Signal task) after physical activity intervention (*r* = 0.22) (96). Using fMRI, Choi et al. found moderate-sized associations between changes in right prefrontal activation and improvements in mental flexibility (shown as decreased preservation errors during Wisconsin card sorting test) and decreased ADHD symptoms after aerobic intervention (*r* = 0.53–0.57) (91). Similarly, Yu et al. found associations between increased EEG N2 amplitude/decreased N2 latency and the improvements in mental flexibility (indicated by increased accuracy during Flanker task; *r* = −0.44–0.46; Supplementary Tables 4, 5) (105). The significant associations between neural and behavioral improvements suggest that the neural measures reflect the underlying neural mechanisms for behavioral improvements and may be used as objective and sensitive measures to assess intervention effects.

## Discussion

### Summary of Main Findings

This review aimed to summarize findings on neurobiomarkers of chronic and acute effects of physical activity/movement intervention using different neuroimaging tools and quantified effect size estimates for various neural outcome measures. Our review of 32 experimental studies revealed that 84% of the studies were fair to good quality (RCT, CCT, or cross-over design studies) and supported the use of neuroimaging techniques, including EEG, fMRI, DTI, and fNIRS, as objective measures for capturing training-related changes in neural processing in individuals with developmental disabilities. Both chronic and acute movement interventions led to positive effects on behavioral measures of social communicational, emotional, and cognitive/executive functions (i.e., inhibitory control, mental flexibility, memory) as well as improved neural function/processing. We found larger effects for chronic movement interventions (Hedges' g = −2.34 to 2.87) compared to acute effects of physical activity (Hedges' g = −1.1 to 1.17). Specifically, movement training led to normalized resting-state, cortical arousal in children with ADHD, normalized resting-state neural connectivity between brain regions important for social communication performance in children with ASD, and normalized neural activity during executive functioning tasks (i.e., tasks involving inhibitory control, memory, and mental flexibility) in individuals with ADHD, ASD, DCD, and LD. Despite the promising results, more research with larger sample sizes and standardized neuroimaging methods across multiple diagnoses is needed to further explore the underlying neural mechanisms and to increase the replicability of findings within and across diagnoses.

### Neural Biomarkers for the Effect of Physical Activity/Movement Intervention in Individuals With Developmental Disabilities

With advances in neuroimaging techniques, more and more intervention studies are including neuroimaging tools as objective outcome measures of intervention effects (58). Systematic reviews involving healthy populations support the use of neuroimaging tools as outcome measures and propose potential mechanisms underlying training-related improvements (73, 79). The current systematic review extends these findings to individuals with developmental disabilities. A large proportion of the studies included in the current systematic review showed significant changes in at least one neural measure after movement intervention (Chronic: 16 out of the 18 included studies; Acute: 12 out of the 15 included studies). Moreover, the training-related changes in neural activity were correlated with behavioral improvements as indicated by a few studies included in the review (91, 96, 105). In short, neuroimaging tools may serve as promising outcome measures to objectively report training effects in individuals with developmental disabilities. Below, we summarize the key findings of the review in terms of neural effects and associated biomarkers of movement interventions in individuals with developmental disabilities.

#### Normalized Resting-State Cortical Arousal and ERP Components During Executive Functioning Tasks in Individuals With Developmental Disabilities

The EEG resting-state fast-wave (i.e., Alpha and Beta band), slow-wave (i.e., Theta band), and their ratios (i.e., Theta/alpha and Theta/Beta ratios) are said to reflect the cortico-subcortical crosstalk/arousal, which in turn affect executive functioning performance (116). Most studies assessing cortical arousal have focused on individuals with ADHD and found reduced resting-state fast-wave activity (i.e., alpha and beta bands), increased slow-wave activity (i.e., Theta band), and increased theta/alpha, theta/beta power ratios in individuals with ADHD compared to healthy individuals (117, 118). The ADHD-related differences in resting-state activity might reflect atypical cortical-subcortical crosstalk/arousal and a lack of inhibition of irrelevant sensory inputs (116, 119). Studies included in the current systematic review found normalized EEG resting-state activity [i.e., increases in alpha power (91, 104), as well as decreases in theta power (91, 97) and theta/alpha (93) and theta/beta ratios (91, 94)] after aerobic physical activity, suggesting normalized cortico-subcortical crosstalk/arousal in children with ADHD.

Apart from resting-state neural activity, several ERP components are said to be reflective of neural processing during executive functioning tasks. For example, greater P3b/N2 amplitudes and shorter P3b/N2 latency indicate more efficient stimuli processing, response monitoring, and memory storage (66). Case-control studies suggested reduced P3b and N2 amplitudes and increased latencies during inhibitory control and mental flexibility tasks in individuals with ASD, ADHD, and/or LD (64–66). Moreover, children with DCD were found to have smaller P3b amplitude during a visuospatial working memory task compared to their TD peers (106). Studies included in the current systematic review found training-related behavioral improvements along with increased P3b amplitude (99, 102, 107, 108), N2 amplitude (95, 96, 105), as well as reduced P3b latency (102, 103, 106) and N2 latency (105, 110) in children with ASD, ADHD, LD, and DCD during inhibitory control, mental flexibility, and working memory tasks. Similar neural mechanisms were found in healthy individuals, with increased P3b amplitude and reduced P3b latency during executive functioning tasks associated with higher fitness levels (74, 75). It is postulated that aerobic exercise may lead to changes in cerebral metabolism, increased blood flow, and the release of neurotransmitters/ neurotrophic factors, such as norepinephrine and dopamine, and serum brain derived neurotrophic factors, leading to changes in cortical arousal which in turn increase the efficacy of stimuli processing, response monitoring, and memory storage during executive functioning tasks (120–122).

#### Increased Social Brain Connectivity in Children With ASD

Children with ASD are known to have abnormalities in cortico-cortical and cortico-subcortical connectivity (123–126). For example, excessive short-range connectivity (prefrontal, temporal, etc.) and reduced long-range connectivity between cortical regions (fronto-parietal, fronto-temporal, etc.) as well as between various cortical and subcortical structures (cortico-cerebellar and cortico-striatal connections) are well-documented in children and adolescents with ASD (123–129). DTI studies have found that children with ASD have lower fractional anisotropy and higher mean diffusivity values in the corpus callosum, internal capsule, fronto-occipital fasciculus, and corticospinal tract, and these differences were associated with their social communication deficits (127, 129). The DTI and fMRI studies in this systematic review reported training-related changes in resting-state neural activity in regions important for social communication performance in children with ASD (85, 89, 90). After 12 weeks of mini-basketball training in children with ASD, training-related improvements in social responsiveness were reported (85, 90). Additionally, using DTI and fMRI, researchers also found normalized white matter integrity (including increased fractional anisotropy in the corpus callosum, fornix, fronto-occipital fasciculus, cerebellar peduncle, internal capsule) and mean diffusivity in the corticospinal tract, as well as increased connectivity between left inferior frontal gyrus and right cerebellum (85, 90). Movement interventions such as mini-basketball training are team sports that require children to set goals, make decisions, take turns, communicate with each other, and manage conflicts in a supportive environment, which in turn, might improve social responsiveness of children with ASD. At a neural level, this may present as increasing efficacy of social/motor information transmission and normalizing of white matter integrity (85).

Similarly, Sharda et al. found reduced resting-state fMRI overconnectivity between the auditory and visual regions and underconnectivity between the auditory and motor regions after 8–12 weeks of music therapy in children with ASD (89). Moreover, the changes in connectivity were associated with improvements in children's communication skills (89). Music and movement interventions/experiences are known to have multisystem and multimodal effects on social, language, and cognitive performance of typically developing children/healthy adults and those with developmental disabilities (49). Musical training involves turn-taking and tuning to the actions of partners during duet/group musical performance which engages the social brain networks in the fronto-temporo-parietal cortices (130, 131). One study found greater fNIRS activation in the temporo-parietal and sensori-motor regions of musicians when they played the second violin part as followers compared to when they played the first violin part as leaders which required greater individual motor planning (132). Such repeated experiences may shape the cortical connectivity of individuals over the long term. DTI measures in musicians with 15 years of experience found reduced diffusivity and greater fiber coherence in effector-specific pathways including corticospinal tracts, superior longitudinal fasciculus, and corpus callosum (133). Additionally, structural MRI studies have widely confirmed that musical training leads to enhancements in the gray and white matter of auditory and effector-specific motor cortices which were in turn associated with musical performance of the participants (134, 135). These findings further confirm the neuroplastic changes following musical training reported by Sharda et al. (89). They postulated an increase in bottom-up sensory processing following music therapy which may contribute to the functional connectivity changes within the auditory and motor cortices. Nevertheless, there is limited literature on cascading social communication effects of physical activity/movement interventions on the neural functioning of individuals with disabilities, and results from this review need to be further confirmed by other studies with larger samples and long-term follow-ups.

#### Increased Functional Activation/Connectivity Within Frontal-Parietal Network During Executive Functioning Tasks in Individuals With Developmental Disabilities

The frontoparietal network, primarily composed of the lateral prefrontal, inferior parietal lobe, and posterior inferior temporal lobes, plays an important role in executive functioning, including inhibitory control, mental flexibility, and memory retrieval (136–139). Specifically, the prefrontal cortex is important for monitoring and sending top-down signals to other cortical/subcortical regions (140); while the parietal regions are particularly important for selective attention whereby the information is selected for preferential processing (136, 139). Case-control studies had found hypoactivation over the frontoparietal network in individuals with ASD and ADHD during executive functioning tasks (41–43, 59). Using fNIRS, Bremer et al. found increased prefrontal cortex activation during inhibitory control tasks after a circuit-based intervention (84). Similarly, fMRI studies found increased connectivity between the left inferior frontal gyrus and right cerebellum, increased parietal activation during inhibitory control, and increased frontal and parietal activation during mental flexibility tasks, following movement interventions (90, 100, 113). Physical activity/movement interventions might benefit executive functioning performance by improving the top-down monitoring and selective attention for stimulus processing.

### Diagnosis-Specific Intervention Program and Related Outcome Measures

Most studies that focused on individuals with ADHD used structured physical activity/aerobic interventions, such as treadmill running and cycling, to promote their executive functioning. Despite some inconsistency, the results generally support the use of physical activity/aerobic interventions to promote executive functioning in individuals with ADHD. Using fMRI and EEG measures, studies suggested normalized resting-state cortical arousal, as well as normalized ERPs and increased activation over the frontoparietal network during executive functioning tasks. Compared to the studies in individuals with ADHD, studies in individuals with ASD have used more multisystem, creative movement interventions (i.e., martial arts, theater, and music and movement interventions) to improve a wide range of skills, including ASD symptoms, social communication skills, and executive functioning. ASD is a multisystem disorder that not only leads to core impairments in social communication skills and repetitive behaviors, but also affects children's motor performance, sensory processing, and cognitive functioning from infancy through adolescence (2–29). Our current review suggests improved sleep quality, social communication skills, executive functioning, as well as enhanced social brain connectivity along with normalized EEG/ERP variables and increased activation over the frontoparietal network during executive functioning tasks. Similar behavioral and neural findings of the effects of physical activity/movement intervention on executive functioning were found in individuals with DCD and LD. Studies of individuals with ID focused on emotional changes and motivation toward physical activity/movement interventions, and found greater motivation to adhere to exercise following an enjoyable badminton training program compared to a treadmill running program. In terms of limitations of the examined literature, the majority of the studies were conducted in school-age children between 6 and 18 years needing less support (i.e., high-functioning children) perhaps, because neuroimaging tools generally require compliance and persistence through testing. Few studies included children with ID, LD, and DCD while the majority assessed intervention effects in children with ADHD/ASD. Lastly, the majority of the studies did not examine follow-up retention effects.

### Limitations

Our effect size calculations might not be representative of all studies investigating neural effects of physical activity/movement interventions because we were only able to calculate effect sizes if the mean and standard deviations of outcome variables were provided by the authors. We also did not include theses and dissertations in our review. Lastly, due to the scarcity of literature on neural effects of movement interventions, we included studies examining effects of various perceptuomotor interventions including multisystem, creative movement (music, dance, etc.), and targeted physical activity (treadmill, cycling, etc.). As discussed earlier, readers should be careful to differentiate when postulating the neural mechanisms of the various movement interventions included in this review. Although multiple cross-over design studies used a counterbalancing approach, they did not report details such as the method of allocation to intervention order or allocation ratio. In general, neuroimaging studies reporting effects on neurobiomarkers post-intervention should comply with CONSORT guidelines when reporting study details (141).

### Implications and Recommendations for Clinical Practice

In terms of the duration of physical activity/movement interventions, our systematic review found larger effects for chronic compared to acute interventions. This is also confirmed by recent reviews and meta-analyses of physical activity interventions in healthy and neurologically affected individuals reporting significant positive effects on working memory after chronic but not acute physical activity interventions (142, 143). Clinicians should recommend longer intervention periods within and across bouts for their clients (i.e., 50 min or more, 1–2 sessions/week, up to 10 weeks or more) to yield better results compared to a single session/shorter periods of physical activity/movement interventions. Weekly consistency and continued physical activity/movement interventions over the long term will likely have a greater positive impact on neural, social, and cognitive functioning. In terms of physical activity/movement intervention types, circuit-based exercise led to greater cognitive/executive functioning improvements compared to continuous treadmill training perhaps, due to the greater cognitive demands of switching between exercises (84). Certain other exercise forms such as badminton training have led to greater exercise adherence suggesting that motivation and enjoyment will be crucial in continuing exercise in the long-term (109, 114). Aerobic exercise (e.g., cycling at 65–70% Heart Rate max) may have more cognitive benefits compared to gentler coordination exercises requiring static and dynamic balance (99, 143). Lastly, after the onset of the COVID-19 pandemic, there has been a rise in use of telehealth as an alternative intervention delivery method. It will be important to understand the differences in behavioral and neural effects of physical activity/movement interventions delivered through virtual vs. traditional, face-to-face approaches (144–147). Further research is needed to understand how different types and delivery methods of physical activity/movement interventions might lead to differential neural effects on social communication and cognitive performance.

### Implications and Recommendation for Future Research

Our review of studies supports the use of different neuroimaging tools as objective measures for intervention effects including MRI/fMRI, DTI, EEG, and fNIRS. The majority of the studies included in the current systematic review used EEG to investigate the movement-related changes in neural activity, probably due to its low-cost and child-friendly nature. EEG-based neurobiomarkers (i.e., slow and fast-wave EEG activity, and the ERPs such as P3b, N2 peaks) could be used to study neural effects of movement interventions on children's networks related to cognitive/executive functioning and social functioning. Besides EEG, other non-invasive, child-friendly techniques include fNIRS (58, 84). Using fNIRS, our research group has reported differences in cortical activation in infants at-risk for and children with ASD during socially embedded actions (i.e., actions performed with adults and caregivers), solo movements, and action observation compared to healthy children and adults during social interaction as well as interpersonal synchrony tasks involving reaching and whole-body movements (67–72). We have consistently found lower fNIRS activation in the superior temporal sulcus and middle/inferior frontal gyri in infants at-risk for and children with ASD compared to controls (67–72). In certain tasks involving synchronous reaching and body sway, fNIRS activation was associated with ASD severity and communication performance (69, 71). Moreover, in an ongoing RCT study, we are investigating the neural effects of creative movement and physical activity/exercise-based movement interventions compared to sedentary, standard of care interventions using fNIRS to track the intervention-related differences during executive functioning and interpersonal synchrony tasks in children with ASD (147). In short, there are alternative, child-friendly approaches robust against motion artifacts that should be considered to study intervention-based changes in neurobiomarkers in individuals with wide-ranging severity in developmental disabilities. Despite the promising results from the studies covered in this review, a lot more remains to be done to develop a deeper understanding of neural mechanisms underlying movement intervention-related improvements. Studies should make it a point to report relationships between changes in neural activity and behavioral performance (imaging task and standard measures). There is little understanding about how certain subgroups based on impairment severity (e.g., level of cognitive or social impairment) and intervention characteristics (e.g., type and intensity of exercise) might have differential impacts on neurobiomarkers. Future studies should include individuals from different subgroups based on age, sex, ethnicity, diagnoses, impairment levels, and use interventions of different types (e.g., aerobic vs. circuit training), intensities (moderate, vigorous, etc.), and durations (30–90 min, etc.) to investigate relations between neural effects and sample/intervention characteristics.

## Conclusion

We conducted a comprehensive review of studies that investigated the neural effects of physical activity/movement interventions in individuals with developmental disabilities. Several intervention-related neurobiomarkers were identified along with behavioral improvements in cognitive and social functioning in individuals with developmental disabilities. Specifically, following movement interventions, individuals with developmental disabilities were found to have normalized resting-state cortical arousal, normalized resting-state social brain connectivity, and changes in neural activity during executive functioning tasks. More research with larger sample sizes and standardized neuroimaging tools is needed to further explore the different neural mechanisms underlying the behavioral effects of physical activity/movement interventions and to increase the replicability of findings across studies.

## Data Availability Statement

The original contributions presented in the study are included in the article/Supplementary Material, further inquiries can be directed to the corresponding author.

## Author Contributions

W-CS, SS, and AB contributed to conception and design of the study. W-CS developed, organized the literature database, and wrote the first draft of the manuscript. SS, CC, and AB assisted with establishing coding reliability. NA and W-CS performed the statistical analysis. SS and AB wrote several sections of the manuscript. All authors contributed to manuscript revision, proof reading, and approved the submitted version.

## Funding

AB's efforts during the writing of this manuscript were supported by a Clinical Neuroscience Award from the Dana Foundation and multiple National Institutes of Health grants (Grant Nos. #: S10-OD021534 and P20-GM103446). SS's efforts during the writing of this manuscript were supported by a Research Excellence program Award from the University of Connecticut and an Institute for Collaboration on Health, Intervention, and Policy (InCHIP) seed grant for faculty affiliates.

## Conflict of Interest

The authors declare that the research was conducted in the absence of any commercial or financial relationships that could be construed as a potential conflict of interest.

## Publisher's Note

All claims expressed in this article are solely those of the authors and do not necessarily represent those of their affiliated organizations, or those of the publisher, the editors and the reviewers. Any product that may be evaluated in this article, or claim that may be made by its manufacturer, is not guaranteed or endorsed by the publisher.
