# Supplementary material for: Neural Effects of Physical Activity and Movement Interventions in Individuals With Developmental Disabilities–A Systematic Review
Source: Front Psychiatry. 2022 Feb 15;13:794652. doi: 10.3389/fpsyt.2022.794652 (PMC8886122; doi:10.3389/fpsyt.2022.794652)
Supplement: Supplementary file 1 [file Data_Sheet_1.docx]

**Supplementary information**

**Table S1. Detailed search terms**

| **Database** | **Search terms** |
| --- | --- |
| **PubMed** | ((Intellectual Disability [Mesh] OR Developmental Disabilities [Mesh] OR Language Development Disorders [Mesh] OR Social Communication Disorder [Mesh] OR Autistic Disorder [Mesh] OR Autism Spectrum Disorder [Mesh] OR Asperger Syndrome [Mesh] OR Child Development Disorders, Pervasive [Mesh] OR Attention Deficit Disorder with Hyperactivity [Mesh] OR Attention Deficit and Disruptive Behavior Disorders [Mesh] OR Motor Skills Disorders [Mesh] OR Down Syndrome [Mesh] OR Learning Disabilities [Mesh] OR Neurodevelopmental disorders[Title/Abstract] OR Neurodevelopmental disorder[Title/Abstract] OR intellectual disabilities[Title/Abstract] OR intellectual disability[Title/Abstract] OR mental retardation[Title/Abstract] OR mentally retarded[Title/Abstract] OR developmental delays[Title/Abstract] OR developmental delay[Title/Abstract] OR developmental disorders[Title/Abstract] OR developmental disorder[Title/Abstract] OR developmental disabilities[Title/Abstract] OR developmental disability[Title/Abstract] OR language disorders[Title/Abstract] OR language disorder[Title/Abstract] OR social communication disorders[Title/Abstract] OR social communication disorder[Title/Abstract] OR social disorders[Title/Abstract] OR social disorder[Title/Abstract] OR communication disorders[Title/Abstract] OR communication disorder[Title/Abstract] OR autism[Title/Abstract] OR autistic[Title/Abstract] OR autism spectrum disorder[Title/Abstract] OR ASD[Title/Abstract] OR ASDs[Title/Abstract] OR Asperger[Title/Abstract] OR Aspergers[Title/Abstract] OR Asperger’s[Title/Abstract] OR Pervasive Developmental Disorders[Title/Abstract] OR Pervasive Developmental Disorder[Title/Abstract] OR PDD-NOS[Title/Abstract] OR PDD[Title/Abstract] OR Attention-deficit/hyperactivity disorder[Title/Abstract] OR Attention deficits[Title/Abstract] OR Attention deficit[Title/Abstract] OR Hyperkinetic[Title/Abstract] OR hyperactivity disorders[Title/Abstract] OR hyperactivity disorder[Title/Abstract] OR ADHD[Title/Abstract] OR ADDH[Title/Abstract] OR Motor Skills Disorders[Title/Abstract] OR Motor skill disorder[Title/Abstract] OR developmental coordination disorder[Title/Abstract] OR DCD[Title/Abstract] OR Down syndrome[Title/Abstract] OR downs syndrome[Title/Abstract] OR Learning disorders [Title/Abstract] OR Learning disorder [Title/Abstract] OR Learning disabilities[Title/Abstract] OR Learning disability [Title/Abstract] OR dyslexia [Title/Abstract]) AND (Sports [Mesh] OR Exercise [Mesh] OR Exercise Movement Techniques [Mesh] OR Physical Education and Training [Mesh] OR Dance Therapy [Mesh] Dancing [Mesh] OR Music Therapy [Mesh] OR Physical Fitness [Mesh] OR Exercise Therapy [Mesh] OR Games, Recreational [Mesh] OR Sports[Title/Abstract] OR Sport[Title/Abstract] OR Baseball[Title/Abstract] OR Basketball[Title/Abstract] OR Bicycling[Title/Abstract] OR Boxing[Title/Abstract] OR Football[Title/Abstract] OR Golf[Title/Abstract] OR Gymnastic[Title/Abstract] OR Hockey[Title/Abstract] OR Tennis[Title/Abstract] OR Running[Title/Abstract] OR Jogging[Title/Abstract] OR Walking[Title/Abstract] OR hiking[Title/Abstract] OR Skating[Title/Abstract] OR Skiing[Title/Abstract] OR Soccer[Title/Abstract] OR Volleyball [Title/Abstract] OR Water Sports[Title/Abstract] OR Water Sport[Title/Abstract] OR Swimming[Title/Abstract] OR Martial arts[Title/Abstract] OR Martial art[Title/Abstract] OR mind-body[Title/Abstract] OR qigong[Title/Abstract] OR Kung fu[Title/Abstract] OR Gong Fu[Title/Abstract] OR Gongfu[Title/Abstract] OR Hap Ki Do[Title/Abstract] OR Judo[Title/Abstract] OR Karate[Title/Abstract] OR Jujitsu[Title/Abstract] OR Tae kwon do[Title/Abstract] OR Aikido[Title/Abstract] OR Wushu[Title/Abstract] OR Tai chi[Title/Abstract] OR Tai ji[Title/Abstract] OR Taijiquan[Title/Abstract] OR Tai Chi Chuan[Title/Abstract] OR Weight-lifting[Title/Abstract] OR Exercise[Title/Abstract] OR Strength[Title/Abstract] OR strengthening[Title/Abstract] OR resistance training[Title/Abstract] OR Endurance[Title/Abstract] OR stretching[Title/Abstract] OR treadmill[Title/Abstract] OR cycling[Title/Abstract] OR Physical activities[Title/Abstract] OR Physical activity[Title/Abstract] OR physical education[Title/Abstract] OR Yoga[Title/Abstract] OR Pilates[Title/Abstract] OR Aerobic[Title/Abstract] OR Dance[Title/Abstract] OR Dancing[Title/Abstract] OR Ballet[Title/Abstract] OR Creative movements[Title/Abstract] OR Creative movement[Title/Abstract] OR music[Title/Abstract] OR Outdoor activities[Title/Abstract] OR Outdoor activity[Title/Abstract] OR Exergaming[Title/Abstract] OR Aquatic[Title/Abstract] OR play behavior[Title/Abstract] OR play behaviors[Title/Abstract] OR play behaviour[Title/Abstract] OR group play[Title/Abstract] OR play therapies[Title/Abstract] OR play therapy[Title/Abstract] OR imaginative play[Title/Abstract] OR social play[Title/Abstract] OR tumble play[Title/Abstract] OR creative play[Title/Abstract] OR physical play[Title/Abstract] OR symbolic play[Title/Abstract] OR fantasy play[Title/Abstract] OR outdoor play[Title/Abstract] OR exploratory play[Title/Abstract] OR object play[Title/Abstract] OR role play[Title/Abstract] OR therapeutic play[Title/Abstract] OR Play[Title])) AND (Neuroimaging [Mesh] OR Cortical Excitability [Mesh] OR Magnetic Resonance Imaging [Mesh] OR Electroencephalography [Mesh] OR Magnetoencephalography [Mesh] OR Brain function[Title/Abstract] OR brain structure[Title/Abstract] OR Neural imaging[Title/Abstract] OR Neuroimaging[Title/Abstract] OR hemodynamics[Title/Abstract] OR Neural effect[Title/Abstract] OR Brain Plasticity[Title/Abstract] OR Neural plasticity[Title/Abstract] OR Neuronal Plasticities[Title/Abstract] OR Neuroplasticity[Title/Abstract] OR Neuroplasticities[Title/Abstract] OR Synaptic Plasticity[Title/Abstract] OR Functional magnetic resonance imaging[Title/Abstract] OR fMRI[Title/Abstract] OR blood oxygen level dependent[Title/Abstract] OR BOLD[Title/Abstract] OR Magnetic Resonance Imaging[Title/Abstract] OR Voxel-based morphometry[Title/Abstract] OR VBM[Title/Abstract] OR MRI[Title/Abstract] OR NMR[Title/Abstract] OR Tomography[Title/Abstract] OR Electroencephalography[Title/Abstract] OR EEG[Title/Abstract] OR Evoked Response Potentials[Title/Abstract] OR ERP[Title/Abstract] OR Functional near-infrared spectroscopy[Title/Abstract] OR near infrared spectroscopy[Title/Abstract] OR FNIRS[Title/Abstract] OR NIRS[Title/Abstract] OR Magnetoencephalography[Title/Abstract] OR MEG[Title/Abstract] OR Diffusion Tensor Imaging[Title/Abstract] OR DTI[Title/Abstract] OR Resting state[Title/Abstract] OR connectivity[Title/Abstract]) |
| **PsycINFO & CINAHL** | (TITLE(“Neurodevelopmental disorders” OR “Neurodevelopmental disorder” OR “intellectual disabilities” OR “intellectual disability” OR “mental retardation” OR “mentally retarded” OR “developmental delays” OR “developmental delay” OR “developmental disorders” OR “developmental disorder” OR “developmental disabilities” OR “developmental disability” OR “language disorders” OR “language disorder” OR “social communication disorders” OR “social communication disorder” OR “social disorders” OR “social disorder” OR “communication disorders” OR “communication disorder” OR “autism” OR “autistic” OR “autism spectrum disorder” OR “ASD” OR “ASDs” OR “Asperger” OR “Aspergers” OR “Asperger’s” OR “Pervasive Developmental Disorders” OR “Pervasive Developmental Disorder” OR “PDD-NOS” OR “PDD” OR “Attention-deficit/hyperactivity disorder” OR “Attention deficits” OR “Attention deficit” OR “Hyperkinetic” OR “hyperactivity disorders” OR “hyperactivity disorder” OR “ADHD” OR “ADDH” OR “Motor Skills Disorders” OR “Motor skill disorder” OR “developmental coordination disorder” OR “DCD” OR “Down syndrome” OR “downs syndrome” OR “Learning disorders” OR “Learning disorder” OR “Learning disabilities” OR “Learning disability” OR “dyslexia”) OR ABS(“Neurodevelopmental disorders” OR “Neurodevelopmental disorder” OR “intellectual disabilities” OR “intellectual disability” OR “mental retardation” OR “mentally retarded” OR “developmental delays” OR “developmental delay” OR “developmental disorders” OR “developmental disorder” OR “developmental disabilities” OR “developmental disability” OR “language disorders” OR “language disorder” OR “social communication disorders” OR “social communication disorder” OR “social disorders” OR “social disorder” OR “communication disorders” OR “communication disorder” OR “autism” OR “autistic” OR “autism spectrum disorder” OR “ASD” OR “ASDs” OR “Asperger” OR “Aspergers” OR “Asperger’s” OR “Pervasive Developmental Disorders” OR “Pervasive Developmental Disorder” OR “PDD-NOS” OR “PDD” OR “Attention-deficit/hyperactivity disorder” OR “Attention deficits” OR “Attention deficit” OR “Hyperkinetic” OR “hyperactivity disorders” OR “hyperactivity disorder” OR “ADHD” OR “ADDH” OR “Motor Skills Disorders” OR “Motor skill disorder” OR “developmental coordination disorder” OR “DCD” OR “Down syndrome” OR “downs syndrome” OR “Learning disorders” OR “Learning disorder” OR “Learning disabilities” OR “Learning disability” OR “dyslexia”)) AND (TITLE(“Sports” OR “Sport” OR “Baseball” OR “Basketball” OR “Bicycling” OR “Boxing” OR “Football” OR “Golf” OR “Gymnastic” OR “Hockey” OR “Tennis” OR “Running” OR “Jogging” OR “Walking” OR “hiking” OR “Skating” OR “Skiing” OR “Soccer” OR “Volleyball” OR “Water Sports” OR “Water Sport” OR “Swimming” OR “Martial arts” OR “Martial art” OR “mind-body” OR “qigong” OR “Kung fu” OR “Gong Fu” OR “Gongfu” OR “Hap Ki Do” OR “Judo” OR “Karate” OR “Jujitsu” OR “Tae kwon do” OR “Aikido” OR “Wushu” OR “Tai chi” OR “Tai ji” OR “Taijiquan” OR “Tai Chi Chuan” OR “Weight-lifting” OR “Exercise” OR “Strength” OR “strengthening” OR “resistance training” OR “Endurance” OR “stretching” OR “treadmill” OR “cycling” OR “Physical activities” OR “Physical activity” OR “physical education” OR “Yoga” OR “Pilates” OR “Aerobic” OR “Dance” OR “Dancing” OR “Ballet” OR “Creative movements” OR “Creative movement” OR “music” OR “Outdoor activities” OR “Outdoor activity” OR “Exergaming” OR “Aquatic” OR “play behavior” OR “play behaviors” OR “play behavior” OR “group play” OR “play therapies” OR “play therapy” OR “imaginative play” OR “social play” OR “tumble play” OR “creative play” OR “physical play” OR “symbolic play” OR “fantasy play” OR “outdoor play” OR “exploratory play” OR “object play” OR “role play” OR “therapeutic play” OR “Play”) OR ABS(“Sports” OR “Sport” OR “Baseball” OR “Basketball” OR “Bicycling” OR “Boxing” OR “Football” OR “Golf” OR “Gymnastic” OR “Hockey” OR “Tennis” OR “Running” OR “Jogging” OR “Walking” OR “hiking” OR “Skating” OR “Skiing” OR “Soccer” OR “Volleyball” OR “Water Sports” OR “Water Sport” OR “Swimming” OR “Martial arts” OR “Martial art” OR “mind-body” OR “qigong” OR “Kung fu” OR “Gong Fu” OR “Gongfu” OR “Hap Ki Do” OR “Judo” OR “Karate” OR “Jujitsu” OR “Tae kwon do” OR “Aikido” OR “Wushu” OR “Tai chi” OR “Tai ji” OR “Taijiquan” OR “Tai Chi Chuan” OR “Weight-lifting” OR “Exercise” OR “Strength” OR “strengthening” OR “resistance training” OR “Endurance” OR “stretching” OR “treadmill” OR “cycling” OR “Physical activities” OR “Physical activity” OR “physical education” OR “Yoga” OR “Pilates” OR “Aerobic” OR “Dance” OR “Dancing” OR “Ballet” OR “Creative movements” OR “Creative movement” OR “music” OR “Outdoor activities” OR “Outdoor activity” OR “Exergaming” OR “Aquatic” OR “play behavior” OR “play behaviors” OR “play behavior” OR “group play” OR “play therapies” OR “play therapy” OR “imaginative play” OR “social play” OR “tumble play” OR “creative play” OR “physical play” OR “symbolic play” OR “fantasy play” OR “outdoor play” OR “exploratory play” OR “object play” OR “role play” OR “therapeutic play” )) AND (TITLE(“Brain function” OR “brain structure” OR “Neural imaging” OR “Neuroimaging” OR “hemodynamics” OR “Neural effect” OR “Brain Plasticity” OR “Neural plasticity” OR “Neuronal Plasticities” OR “Neuroplasticity” OR “Neuroplasticities” OR “Synaptic Plasticity” OR “Functional magnetic resonance imaging” OR “fMRI” OR “blood oxygen level dependent” OR “BOLD” OR “Magnetic Resonance Imaging” OR “Voxel-based morphometry” OR “VBM” OR “MRI” OR “NMR” OR “Tomography” OR “Electroencephalography” OR “EEG” OR “Evoked Response Potentials” OR “ERP” OR “Functional near-infrared spectroscopy” OR “near infrared spectroscopy” OR “FNIRS” OR “NIRS” OR “Magnetoencephalography” OR “MEG” OR “Diffusion Tensor Imaging” OR “DTI” OR “Resting state” OR “connectivity” OR “default mode network”) OR ABS(“Brain function” OR “brain structure” OR “Neural imaging” OR “Neuroimaging” OR “hemodynamics” OR “Neural effect” OR “Brain Plasticity” OR “Neural plasticity” OR “Neuronal Plasticities” OR “Neuroplasticity” OR “Neuroplasticities” OR “Synaptic Plasticity” OR “Functional magnetic resonance imaging” OR “fMRI” OR “blood oxygen level dependent” OR “BOLD” OR “Magnetic Resonance Imaging” OR “Voxel-based morphometry” OR “VBM” OR “MRI” OR “NMR” OR “Tomography” OR “Electroencephalography” OR “EEG” OR “Evoked Response Potentials” OR “ERP” OR “Functional near-infrared spectroscopy” OR “near infrared spectroscopy” OR “FNIRS” OR “NIRS” OR “Magnetoencephalography” OR “MEG” OR “Diffusion Tensor Imaging” OR “DTI” OR “Resting state” OR “connectivity” OR “default mode network”)) |
| **Scopus** | (TITLE(“Neurodevelopmental disorders” OR “Neurodevelopmental disorder” OR “intellectual disabilities” OR “intellectual disability” OR “mental retardation” OR “mentally retarded” OR “developmental delays” OR “developmental delay” OR “developmental disorders” OR “developmental disorder” OR “developmental disabilities” OR “developmental disability” OR “language disorders” OR “language disorder” OR “social communication disorders” OR “social communication disorder” OR “social disorders” OR “social disorder” OR “communication disorders” OR “communication disorder” OR “autism” OR “autistic” OR “autism spectrum disorder” OR “ASD” OR “ASDs” OR “Asperger” OR “Aspergers” OR “Asperger’s” OR “Pervasive Developmental Disorders” OR “Pervasive Developmental Disorder” OR “PDD-NOS” OR “PDD” OR “Attention-deficit/hyperactivity disorder” OR “Attention deficits” OR “Attention deficit” OR “Hyperkinetic” OR “hyperactivity disorders” OR “hyperactivity disorder” OR “ADHD” OR “ADDH” OR “Motor Skills Disorders” OR “Motor skill disorder” OR “developmental coordination disorder” OR “DCD” OR “Down syndrome” OR “downs syndrome” OR “Learning disorders” OR “Learning disorder” OR “Learning disabilities” OR “Learning disability” OR “dyslexia”) OR ABS(“Neurodevelopmental disorders” OR “Neurodevelopmental disorder” OR “intellectual disabilities” OR “intellectual disability” OR “mental retardation” OR “mentally retarded” OR “developmental delays” OR “developmental delay” OR “developmental disorders” OR “developmental disorder” OR “developmental disabilities” OR “developmental disability” OR “language disorders” OR “language disorder” OR “social communication disorders” OR “social communication disorder” OR “social disorders” OR “social disorder” OR “communication disorders” OR “communication disorder” OR “autism” OR “autistic” OR “autism spectrum disorder” OR “ASD” OR “ASDs” OR “Asperger” OR “Aspergers” OR “Asperger’s” OR “Pervasive Developmental Disorders” OR “Pervasive Developmental Disorder” OR “PDD-NOS” OR “PDD” OR “Attention-deficit/hyperactivity disorder” OR “Attention deficits” OR “Attention deficit” OR “Hyperkinetic” OR “hyperactivity disorders” OR “hyperactivity disorder” OR “ADHD” OR “ADDH” OR “Motor Skills Disorders” OR “Motor skill disorder” OR “developmental coordination disorder” OR “DCD” OR “Down syndrome” OR “downs syndrome” OR “Learning disorders” OR “Learning disorder” OR “Learning disabilities” OR “Learning disability” OR “dyslexia”)) AND (TITLE(“Sports” OR “Sport” OR “Baseball” OR “Basketball” OR “Bicycling” OR “Boxing” OR “Football” OR “Golf” OR “Gymnastic” OR “Hockey” OR “Tennis” OR “Running” OR “Jogging” OR “Walking” OR “hiking” OR “Skating” OR “Skiing” OR “Soccer” OR “Volleyball” OR “Water Sports” OR “Water Sport” OR “Swimming” OR “Martial arts” OR “Martial art” OR “mind-body” OR “qigong” OR “Kung fu” OR “Gong Fu” OR “Gongfu” OR “Hap Ki Do” OR “Judo” OR “Karate” OR “Jujitsu” OR “Tae kwon do” OR “Aikido” OR “Wushu” OR “Tai chi” OR “Tai ji” OR “Taijiquan” OR “Tai Chi Chuan” OR “Weight-lifting” OR “Exercise” OR “Strength” OR “strengthening” OR “resistance training” OR “Endurance” OR “stretching” OR “treadmill” OR “cycling” OR “Physical activities” OR “Physical activity” OR “physical education” OR “Yoga” OR “Pilates” OR “Aerobic” OR “Dance” OR “Dancing” OR “Ballet” OR “Creative movements” OR “Creative movement” OR “music” OR “Outdoor activities” OR “Outdoor activity” OR “Exergaming” OR “Aquatic” OR “play behavior” OR “play behaviors” OR “play behavior” OR “group play” OR “play therapies” OR “play therapy” OR “imaginative play” OR “social play” OR “tumble play” OR “creative play” OR “physical play” OR “symbolic play” OR “fantasy play” OR “outdoor play” OR “exploratory play” OR “object play” OR “role play” OR “therapeutic play” OR “Play”) OR ABS(“Sports” OR “Sport” OR “Baseball” OR “Basketball” OR “Bicycling” OR “Boxing” OR “Football” OR “Golf” OR “Gymnastic” OR “Hockey” OR “Tennis” OR “Running” OR “Jogging” OR “Walking” OR “hiking” OR “Skating” OR “Skiing” OR “Soccer” OR “Volleyball” OR “Water Sports” OR “Water Sport” OR “Swimming” OR “Martial arts” OR “Martial art” OR “mind-body” OR “qigong” OR “Kung fu” OR “Gong Fu” OR “Gongfu” OR “Hap Ki Do” OR “Judo” OR “Karate” OR “Jujitsu” OR “Tae kwon do” OR “Aikido” OR “Wushu” OR “Tai chi” OR “Tai ji” OR “Taijiquan” OR “Tai Chi Chuan” OR “Weight-lifting” OR “Exercise” OR “Strength” OR “strengthening” OR “resistance training” OR “Endurance” OR “stretching” OR “treadmill” OR “cycling” OR “Physical activities” OR “Physical activity” OR “physical education” OR “Yoga” OR “Pilates” OR “Aerobic” OR “Dance” OR “Dancing” OR “Ballet” OR “Creative movements” OR “Creative movement” OR “music” OR “Outdoor activities” OR “Outdoor activity” OR “Exergaming” OR “Aquatic” OR “play behavior” OR “play behaviors” OR “play behavior” OR “group play” OR “play therapies” OR “play therapy” OR “imaginative play” OR “social play” OR “tumble play” OR “creative play” OR “physical play” OR “symbolic play” OR “fantasy play” OR “outdoor play” OR “exploratory play” OR “object play” OR “role play” OR “therapeutic play” )) AND (TITLE(“Brain function” OR “brain structure” OR “Neural imaging” OR “Neuroimaging” OR “hemodynamics” OR “Neural effect” OR “Brain Plasticity” OR “Neural plasticity” OR “Neuronal Plasticities” OR “Neuroplasticity” OR “Neuroplasticities” OR “Synaptic Plasticity” OR “Functional magnetic resonance imaging” OR “fMRI” OR “blood oxygen level dependent” OR “BOLD” OR “Magnetic Resonance Imaging” OR “Voxel-based morphometry” OR “VBM” OR “MRI” OR “NMR” OR “Tomography” OR “Electroencephalography” OR “EEG” OR “Evoked Response Potentials” OR “ERP” OR “Functional near-infrared spectroscopy” OR “near infrared spectroscopy” OR “FNIRS” OR “NIRS” OR “Magnetoencephalography” OR “MEG” OR “Diffusion Tensor Imaging” OR “DTI” OR “Resting state” OR “connectivity” OR “default mode network”) OR ABS(“Brain function” OR “brain structure” OR “Neural imaging” OR “Neuroimaging” OR “hemodynamics” OR “Neural effect” OR “Brain Plasticity” OR “Neural plasticity” OR “Neuronal Plasticities” OR “Neuroplasticity” OR “Neuroplasticities” OR “Synaptic Plasticity” OR “Functional magnetic resonance imaging” OR “fMRI” OR “blood oxygen level dependent” OR “BOLD” OR “Magnetic Resonance Imaging” OR “Voxel-based morphometry” OR “VBM” OR “MRI” OR “NMR” OR “Tomography” OR “Electroencephalography” OR “EEG” OR “Evoked Response Potentials” OR “ERP” OR “Functional near-infrared spectroscopy” OR “near infrared spectroscopy” OR “FNIRS” OR “NIRS” OR “Magnetoencephalography” OR “MEG” OR “Diffusion Tensor Imaging” OR “DTI” OR “Resting state” OR “connectivity” OR “default mode network”)) |

**Table S2. PEDro scale and NIH-ROB scoring criteria for risk of bias analyses.**

| **NO** | **Pedro Scale Criteria** | **Yes=1; No =0** | **Included in total score calculation (X = not included; O = included)** |
| --- | --- | --- | --- |
| 1 | Eligibility criteria were specified |  | X |
| 2 | Subjects were randomly allocated to groups (in a crossover study, subjects were randomly allocated an order in which treatments were received) |  | O |
| 3 | Allocation was concealed |  | O |
| 4 | The groups were similar at baseline regarding the most important prognostic indicators |  | O |
| 5 | There was blinding of all subjects |  | O |
| 6 | There was blinding of all therapists who administered the therapy |  | O |
| 7 | There was blinding of all assessors who measured at least one key outcome |  | O |
| 8 | Measures of at least one key outcome were obtained from more than 85% of the subjects initially allocated to groups |  | O |
| 9 | All subjects for whom outcome measures were available received the treatment or control condition as allocated or, where this was not the case, data for at least one key outcome was analyzed by “intention to treat” |  | O |
| 10 | The results of between-group statistical comparisons are reported for at least one key outcome |  | O |
| 11 | The study provides both point measures and measures of variability for at least one key outcome |  | O |
| Total Score | |  | |
| **NO** | **NIH-ROB Criteria** | **Yes=1; No =0** | **Included in total score calculation (X = not included; O = included)** |
| 1 | Was the study question or objective clearly stated? |  | O |
| 2 | Were eligibility/selection criteria for the study population prespecified and clearly described? |  | O |
| 3 | Were the participants in the study representative of those who would be eligible for the test/service/intervention in the general or clinical population of interest? |  | O |
| 4 | Were all eligible participants that met the prespecified entry criteria enrolled? |  | O |
| 5 | Was the sample size sufficiently large to provide confidence in the findings? |  | O |
| 6 | Was the test/service/intervention clearly described and delivered consistently across the study population? |  | O |
| 7 | Were the outcome measures prespecified, clearly defined, valid, reliable, and assessed consistently across all study participants? |  | O |
| 8 | Were the people assessing the outcomes blinded to the participants' exposures/interventions? |  | O |
| 9 | Was the loss to follow-up after baseline 20% or less? Were those lost to follow-up accounted for in the analysis? |  | O |
| 10 | Did the statistical methods examine changes in outcome measures from before to after the intervention? Were statistical tests done that provided *p* values for the pre-to-post changes? |  | O |
| 11 | Were outcome measures of interest taken multiple times before the intervention and multiple times after the intervention (i.e., did they use an interrupted time-series design)? |  | O |
| 12 | If the intervention was conducted at a group level (e.g., a whole hospital, a community, etc.) did the statistical analysis take into account the use of individual-level data to determine effects at the group level? |  | O |
| Total score | |  | |

**Table S3. Coding form for data extraction of the included studies**

| **Coder** |  | **Date** |  |
| --- | --- | --- | --- |
| **Study Characteristics** | | | |
| **Study identifier** |  | **Author list** |  |
| **Year of publication** |  | **Full citation** | (In APA 6^th^ edition format) |
| **Source of study** | (1= journal, 2 = conference paper) | **Location of study** |  |
| **Year of data collection** |  |  |  |
| **Methodological quality of studies** | | | |
| **Study design** | (1 = RCT, 2 = CCT, 3 = Pre-posttest, 4 = Cross-over) | **Study design** | (1 = between-subject; 2 = within-subject design; 3 = both) |
| **Pedro score** |  | **NIH-ROB score** |  |
| **Type of control group** | (1 = random assignment of individuals; 2 = matching individuals on some variable before random assignment; 3 = tried to ensure some comparability of the non-equivalent control group; 4 = non-equivalence of comparison group was not addressed; 5 = no control group (pre-post)) | | |
| **# of Follow up testing** |  | **Intervals between follow-up** |  |
| **Total sample characteristics** | | | |
| **Total sample size** |  | **Age in subjects** | (Mean (SD) and range) |
| **Diagnosis of the subject** | (ASD, ADHD, ID, DS, DD, DCD, DYS, LD, COMD, LANGD) | **Measures used to establish diagnosis** | (1 = observational measures/interview (CARS, ADI-R), 2 = parent report/ questionnaire; 3 = physician using DSM or ICD, NS) |
| **Sex** | (# of males and females) | **Level of functioning** | (1 = low, 2 = high, NS) |
| **IQ** | (Mean (SD) and range) | **Inclusion of children with IQ score < 70** | (0 = no; 1 = yes) |
| **Socioeconomic Status** |  | **Ethnicity** | (B = Black; M = Multiracial; AI = American Indian/Alaskan Native; NH = Native Hawaiian or other Pacific Islander; W = White; A = Asian; O = other; NS) |
| **Experimental group characteristics** | | | |
| **Sample size** |  | **Age in subjects** | (Mean (SD) and range) |
| **Diagnosis of the subject** | (ASD, ADHD, ID, DS, DD, DCD, DYS, LD, COMD, LANGD) | **Measures used to establish diagnosis** | (1 = observational measures/interview (CARS, GARS, ADI-R), 2 = parent report/ questionnaire; 3 = physician using DSM or ICD, NS) |
| **Sex** | (# of males and females) | **Level of functioning** | (1 = low, 2 = high, NS) |
| **IQ** | (Mean (SD) and range) | **Inclusion of children with IQ score < 70** | (0 = no; 1 = yes) |
| **Socioeconomic Status** |  | **Ethnicity** | (B = Black; M = Multiracial; AI = American Indian/Alaskan Native; NH = Native Hawaiian or other Pacific Islander; W = White; A = Asian; O = other; NS) |
| **Control group characteristics** | | | |
| **Total sample size** |  | **Age in subjects** | (Mean (SD) and range) |
| **Diagnosis of the subject** | (ASD, ADHD, ID, DS, DD, DCD, DYS, LD, COMD, LANGD) | **Measures used to establish diagnosis** | (1 = observational measures/interview (CARS, GARS, ADI-R), 2 = parent report/ questionnaire; 3 = physician using DSM or ICD, NS) |
| **Sex** | (# of males and females) | **Level of functioning** | (1 = low, 2 = high, NS) |
| **IQ** | (Mean (SD) and range) | **Inclusion of children with IQ score < 70** | (0 = no; 1 = yes) |
| **Socioeconomic Status** |  | **Ethnicity** | (B = Black; M = Multiracial; AI = American Indian/Alaskan Native; NH = Native Hawaiian or other Pacific Islander; W = White; A = Asian; O = other; NS) |
| **Experimental group intervention characteristics** | | | |
| **Type of intervention** | (1 = sport/ physical activity, 2 = dance, 3 = music, 3 = yoga, 4 = martial art, 5 = theater, 6 = mind body) | **Name of the intervention** |  |
| **Descriptions of training movement/ content** |  | | |
| **Target skills in the intervention** | (1 = physical activity, 2 = motor skills, 3 = social communication; 4 = mindfulness; 5 = Behavioral; 6 = Cognition; 7 = other) | **Intervention format** | (1 = 1 to 1; 2 = group; 3 = both) |
| **Provider** | (1= teacher; 2 = parent; 3 = licensed clinician; 4 = certified instructor; 5 = not certified staff/paraprofessional) | **Acute or Chronic** | (0 = Acute; 1 = Chronic) |
| **Frequency** | (# of sessions/ week; NA for acute exercise) | **Intensity** | (Reserved HR (%); averaged HR) |
| **Time per session** | (minutes) | **Duration** | (# of weeks) |
| **Home program content** |  | **Home program provider** | (0 = self; 1 = parents; 2 = others) |
| **Control group intervention characteristics** | | | |
| **Type of intervention** | (1 = waitlist; 2 = treatment as usual; 3 = parent education; 4 = other) | **Name of the intervention** |  |
| **Descriptions of training movement/ content** |  | | |
| **Target skills in the intervention** | (1 = physical activity, 2 = motor skills, 3 = social communication; 4 = mindfulness; 5 = Behavioral; 6 = Cognition; 7 = other) | **Intervention format** | (1 = 1 to 1; 2 = group; 3 = both) |
| **Provider** | (1= teacher; 2 = parent; 3 = licensed clinician; 4 = certified instructor; 5 = not certified staff/paraprofessional) | **Acute or Chronic** | (0 = Acute; 1 = Chronic) |
| **Frequency** | (# of sessions/ week; NA for acute exercise) | **Intensity** | (Reserved HR (%); averaged HR) |
| **Time per session** | (minutes) | **Duration** | (# of weeks) |
| **Home program content** |  | **Home program provider** | (0 = self; 1 = parents; 2 = others) |
| **Neural assessments** | | | |
| **Type of neuroimaging tool** | (fMRI, SMRI, fNIRS, EEG, MEG, DTI, TMS) | **Neural imaging system** | (Name of the company, system description) |
| **System coverage** | (1 = whole brain; 2 = partial; 3- unclear) | **Region of interest** |  |
| **Neuroimaging task** | (Resting state; Inhibitory control; Mental flexibility; Memory, etc.) | **Name of the task** |  |
| **Conditions** |  | **# of trials** |  |
| **Variables** | (ERPs, BOLD signals, etc.) | **Statistical analysis** | (1 = nonparametric; 2 = correlation; 3 = t-test and z-test; 4 = ANOVA; 5 = MANOVA/MANCOVA; 6 = regression) |
| **Brief results** |  | **Reported effect sizes** |  |
| **Behavioral assessments** | | | |
| **Types of variables** | (1 = social; 2 = communication; 3 = behavioral problems; 4 = sensory problems; 5 = motor skills; 6 = physical fitness/ activity; 7 = quality of life; 8 = self-care skills; 9 = cognition, 10 = other) | **# of variables** |  |
| **Name of the assessment tool** |  | **Type of the assessment tool** | (1 = standardized scale, 2 = observational measure, 3 = video coding, 4 = questionnaires, 5 = motion tracking system, 6 = computerized, 7 = other) |
| **Variables** | (ERPs, BOLD signals, etc.) | **Statistical analysis** | (1 = nonparametric; 2 = correlation; 3 = t-test and z-test; 4 = ANOVA; 5 = MANOVA/MANCOVA; 6 = regression) |
| **Brief results** |  | **Reported effect sizes** |  |

APA = American Psychological Association; RCT = Randomized Controlled Trial; CCT = Controlled Clinical Trial; SD = Standard Deviation; ASD = Autism Spectrum Disorder; ADHD = Attention-Deficit/Hyperactivity Disorder; ID = Intellectual Disabilities; DS = Down Syndrome; DD = Developmental Delay; DCD = Developmental Coordination Disorder; DYS = Dyslexia; COMD = Communication Disorder; LANGD = Language Disorder; CARS = Childhood Autism Rating Scale; ADI-R = Autism Diagnostic Interview, Revised; DSM = Diagnostic and Statistical Manual of Mental Disorders; ICD = International Classification of Diseases; fMRI = Functional Magnetic Resonance Imaging; sMRI = Structural Magnetic Resonance Imaging; fNIRS = Functional near-infrared spectroscopy; EEG = Electroencephalogram; MEG = Magnetoencephalography; DTI = Diffusion tensor imaging; TMS = Transcranial magnetic stimulation; ERP = Event-related potential; BOLD = Blood oxygenation level-dependent; ANOVA = Analysis of variance; MANOVA = Multiple analysis of variance; MANCOVA = Multivariate Analysis of Covariance; NS = not specified; NA = not applicable.

**Table S4. Main results of the chronic effect studies**

| Study, Year | NeurOimaGing task/ tool | NeuroimaGing findings | behavioral findings | Correlation findings | |
| --- | --- | --- | --- | --- | --- |
| Autism Spectrum Disorders | | | | | |
| BRA, 2015 | Sleep/  EEG | **W**: Sleep EEG variables, including total sleeping time, etc.: Post ≈ Pre | **W**: Sleep quality (Insomnia severity index) & mood: ≈; Ball skills & balance: ↑ | - | |
| CAI, 2020 | Resting-state/  DTI | **W&B:** FA in corpus callosum, fornix, cerebral peduncle, internal capsule, L anterior corona radiates, L superior fronto-occipital fasciculus: Post > pre; E > C; MD in Bil corticospinal tracts, and L anterior corona radiate: Post > Pre; E > C | **W:** Social responsiveness (SRS): ↑ | Children who had lower social cognition performance (SRS) showed greater training-related changes in white matter integrity | |
| Cha, 2013 | Go-no-Go/  EEG | **W**(No-Go condition): theta activation over anterior cingulate cortex: Post > Pre | **W**: Self-control (ToL, CCTT, FPT): ↑; Behavior (parent rating): ↑; Autism related performance: ↑ | - | |
| Cha, 2015 | Visual memory/ EEG | **W: Randomized**- Theta source activity over Bil prefrontal, L parietal and temporal **&** Theta coherence over L frontoposterior: Post > Pre; **Organized:** Theta source activity over Bil prefrontal, parietal, and temporal **&** and Theta coherence over Bil intra-frontoposterior and inter-frontoposterior: Post > Pre | **W: Randomized-** memory (sematic cluster score): ↑; **Organized-** memory (total recall & visual scanning score): ↑ | - | |
| Cor, 2016 | Face memory task/ EEG | **W&B:** ERP amp over parietal: Post > Pre; E > C | **W:** Memory of face (immediate & delayed) & adaptive behaviors: ↑; **B:** Memory of face (delayed), ToM, adaptive behaviors, social responsiveness, group play: ↑ | - | |
| LAG, 2019 | Sensory gating/ EEG | **W:** P50 and N100 difference scores (differences between peak-to-peak amplitude between 1^st^ & 2^nd^ click) over Cz: Post ≈ Pre | **W:** Selective attention: ↑; Switching, sustained, overall attention: ≈ | **Pre intervention:** P50 difference scores **&** sustained attention (r = 0.77); N100 difference score **&** selective attention (r = -0.78) | |
| ShA, 2018 | Resting-state/  fMRI | **B**: Connectivity between auditory and subcortical and motor regions: E > C; Connectivity between auditory and visual regions: E < C | **W:** Adaptive functions: ↑  **B:** Communication skill: ↑ | **Post intervention:** Greater communication skill is associated with increased connectivity between auditory & subcortical regions and decreased connectivity between auditory & visual regions | |
| YAN, 2021 | Resting-state/  fMRI | **W**: Connectivity between left inferior frontal gyrus and right cerebellum: Post > Pre | **W**: Social cognition: ↑ | **Intervention effect:** Differences in fMRI connectivity was not correlated to social performance | |
| Attention-Deficit/Hyperactivity disorder | | | | | |
| CHO, 2015 | Wisconsin Card Sorting Test /  fMRI | **W:** R frontal, L parietal: Post > Pre; R temporal lobe: Post < Pre  **B:** R frontal: E > C | **B::** Mental flexibility (preservation error): ↑; ADHD-related performance (K-ARS total score): ↑ | **Intervention effect:** Increased activity over R prefrontal cortex was associated with improved ADHD-related performance (K-ARS, r = -0.57) and mental flexibility (perseverative errors, r = -0.53) | |
| Hua, 2017 | Resting-state/  EEG | **B:** Theta/alpha ratios over frontal (F3, F4, Fz) and central regions (C3, C4, Cz): E < C | - |  | |
| Jan, 2016A | Stop sign task/  EEG | **W:** N2 amp over Fz, Cz, Pz: Post > Pre  **B:** P3 amp over Fz, Cz, Pz: C > E | - | **Pre intervention:** Larger P3 amp over Cz was associated with better inhibitory control (reaction time, r =  -0.566)  **Intervention effect- E&C:** training-related increase of N2 amp over Cz was associated with the improvements in inhibitory control (reaction time, r = -0.284) | |
| Jan, 2016B | Resting & Stop sign task/ EEG | **B**- **resting**: Mean power reduction of Theta frequency over Fz, Cz, Pz: C > E  **B- Stop sign task**: Mean power reduction of Theta and Alpha frequency over Fz, Cz, Pz: C > E | - | **Pre intervention:** Theta power during resting and stop sign task was associated with hyperactivity (teacher-rated inattention hyperactivity, r = 0.33 to 0.39); Alpha power during resting is associated attention (parent-reported inattentive, r = -0.22) and inhibitory control (errors during stop sign task, r = 0.22) | |
| Lee, 2017 | Resting & Stroop color and word test/ EEG | **W&B-resting:** Beta wave over F3 and F4: Post ≈ Pre; E ≈ C  **W&B-Stroop:**  Beta wave over F3: Post > Pre, E > C; Beta wave over F4: Post > Pre, E ≈ C | **W:** Mental flexibility -Color word score: ↑; **B**: Mental flexibility (Color word and interference scores): ≈ | - | |
| SMI, 2019 | Go-no-Go/  EEG | **B:** N2 amp, N2 latency, P3 amp over frontal central and posterior parietal: E ≈ C; P3 latency over frontal central and posterior parietal: E < C | **B:** Inhibitory control (accuracy and reaction time): ↑≈ | - |  |
| Developmental coordination disorder | | | | | |
| TSA, 2012 | Visuospatial attention/  EEG | **B:** P3 latency over occipital, parietal, temporal regions: E < C; N2 latency, N2 amp, P3 amp over occipital, parietal, temporal regions: E ≈ C | **B:** Attention: error rate: ≈; reaction time: ↑; Motor skills (M-ABC): ↑ | - |  |
| TSA, 2014 | Visuospatial working memory/ EEG | **B:** 1^st^ & 2^nd^ Stimuli evoked P3 amp over frontal, central, temporal, parietal and occipital regions: E > C | **B:** Working memory (respond accuracy): ↑; Cardiorespiratory fitness: ↑; Motor skills (M-ABC): ↑ | - |  |
| Learning disabilities | | | | | |
| Mil, 2019 | Flanker and selective auditory attention/ EEG | **B:** P3 amp during Flanker incongruent: E > C; P3 amp & latency during Flanker congruent & attention tasks: E ≈ C | **B:** Inhibitory control (Response accuracy & reaction time during Flanker and attention tasks): ≈; attention (Conner3 inattentive subscale): ↑ | - | |
| Intellectual disabilities | | | | | |
| CHE, 2021 | Resting-state/ EEG | **W**: Frontal alpha asymmetry: Post > Pre | **W:** Endurance (6 min walk test): ↑, Badminton skill: ↑ Post > Pre; Cardio capacity (resting heart rate): ↑ | - |  |

**W:** results from the within group comparisons of the experimental group between pre & post-tests; **B:** Results from the between group comparisons of pre-test adjusted/unadjusted post-test scores and/or differences between pre-and post-tests; **E**: experimental group; **C**: Comparison group; **EEG**: Electroencephalogram; **DTI**: Diffusion Tensor Imaging; **fMRI**: Functional magnetic resonance imaging; **FA**: Fractional Anisotropy; **MD**: Mean Diffusivity; **SRS**: Social responsiveness scale; **ATEC**: autism treatment evaluation checklist, **ToL**: Tower of London, **CCTT**: children’s color trials test; **FPT**: Five-point test; **K-ARS**: Dual attention deficit hyperactivity disorder rating scale-Korean version; **ToM**: Theory of mind; **Pre**: the neuroimaging findings and/or behavioral performance during pre-test/baseline; **Post**: the neuroimaging findings and/or behavioral performance during post-test/after intervention; **L**: left; **R**: Right; **Bil**: Bilateral; ↑: improved performance after intervention (Within-group comparison) or better performance in the experimental compared to the control group (Between-group comparison); ≈ : no significant within and between groups differences.

**Table S5. Main results of the acute effect studies**

| Study, Year | Neuraoimaing tasks/ tools | Neuroimaing findings | Behavioral findings | Correlation findings |  |
| --- | --- | --- | --- | --- | --- |
| Autism Spectrum Disorder | | | | |  |
| BRA, 2015 | Sleep/ EEG | **B:** Sleep EEG, including total sleep time: Post > Pre | - | - |  |
| BRE, 2020 | Attention sustained subtest of the Leiter International Performance Scale/ fNIRS | **W-Circuit:** Oxy-Hb in prefrontal cortex: Post > Pre  **W-Treadmill:** ≈ | **W-Circuit:** Attention (response accuracy): ↑  **W-Treadmill:** Attention **(**response accuracy): ≈  **W&B- Circuit & Treadmill:** Affect, perceived mental exertion, motivation, self-efficacy: ≈ | - |  |
| Attention-Deficit/Hyperactivity disorder | | | | |  |
| CHO, 2016 | Resting state/ EEG | **W**: Absolute theta in left frontal, relative theta along midline: Post < Pre; absolute alpha in the central and posterior, relative alpha globally: Post > Pre | - | - | |
| CHU, 2021 | Resting state/EEG | **B:** Frontal lobe asymmetry: E1 > E2 > C | - | Increased frontal lobe asymmetry is associated with decreased internalized problems (r = -0.437) | |
| Hua, 2018 | Resting state/ EEG | **W&B:** Theta/Beta ratio over Fz, Cz, Pz: Post < Pre; E < C | - | - | |
| HUN, 2016 | Task switching paradigm/ EEG | P3 amp over Fz, Cz, Pz: Mix condition > Pure condition | **B:** Mental flexibility **(**shift cost of reaction time (mix-pure)): ↑ | - | |
| LUD, 2017 | Modified Flanker task/ EEG | **W&B:** P3 amp over parietal regions: Post > Pre; E > C | **W&B:** Inhibitory control (reaction time): ↑ | - | |
| Meh, 2019A | Flanker & Visual attention task/ fMRI | **B-Flanker task:**  E ≈ C  **B-Visual attention task:** R superior frontal gyrus and anterior frontopolar part of the prefrontal cortex: E < C | **B-Flanker task:** Inhibitory control **(**reaction time): ↑ | Individual with better aerobic capacity (VO_2peak_) showed greater training related improvement in inhibitory control (reaction time) (r = 0.233) | |
| Meh, 2019B | Go-no Go task/ fMRI | **B:** Bil occipital, temporal, and parietal regions: E > C | **B:** Inhibitory control (response accuracy and reaction time): ≈ | Greater activation over supramarginal gyrus, superior temporal gyrus, and rolandic operculum is associated with better inhibitory control (accuracy) (r^2^ = 0.288) | |
| PON, 2013 | Flanker task/ EEG | **B:** P3 amp: E > C; P3 latency over FC, Cz, and CPz: E < C; ERN amp: E ≈ C | **B:** Inhibitory control (response accuracy): ↑ Reading comprehension and arithmetic: ↑ | - | |
| TSA, 2021 | Resting state & Flanker/ EEG | **Resting-W:** E1, Alpha power: Post > Pre**;**  **B:** Alpha power: E1 > E2 > E3  **Flanker:** congruency effects of P3 latency found in E1 and E3 but not E2 | **Flanker:** Response accuracy: E3 ≈ E2 ≈ E1; Reaction time: E1 & E2 < E3 | **Flanker:** In E2, Greater increase in alpha power is associated with greater improvement in inhibitory control (reaction time) | |
| Yu, 2020 | Flanker task/ EEG | **B:** N2 amp over Fz, FCz, Cz: E > C; N2 latency over Fz, FCz, Cz: E < C; P3 amp & latency over CPz & Pz: E ≈ C | **B:** Inhibitory control (response accuracy): ↑ | Increased N2 amplitude and decreased N2 latency is associated with greater improvement in inhibitory control (response accuracy) (r = -0.44& -0.46) | |
| Intellectual disabilities | | | | |  |
| CHE, 2016 | Resting state/ EEG | **W:** Frontal alpha asymmetry: Pre ≈ Post | - | Individuals with greater rate of perceived exertion has less training related changes in frontal asymmetry | |
| VOG, 2012 | Resting state/ EEG | **W**: Cortical current density in rectal gyrus, medial frontal gyrus, orbital gyrus, and Brodmann area 11&25: Post < Pre | **W**: Mood & motivation: ↑; cognition (accuracy & reaction time): ≈ | - | |
| VOG, 2013 | Resting state & Decision making/ EEG | **W (decision making)**: Current density over medial frontal gyrus during resting state **&** N2 latency over frontal lobe: Post < Pre; N2 amp over frontal lobe: Pre ≈ Post | **W**: Decision making (reaction time): ↑ | - | |

**W:** results from the within group comparisons of the experimental group between pre & post-tests; **B:** Results from the between group comparisons of pre-test adjusted/unadjusted post-test scores and/or differences between pre-and post-tests; **E**: experimental group (E1= the first experimental group; E2 = the second experimental group; E3 = the third experimental group); **C**: Comparison group. **EEG**: Electroencephalogram; **fNIRS**: functional near infrared spectroscopy; **fMRI**: Functional magnetic resonance imaging; **Pre:** the neuroimaging findings and/or behavioral performance during pre-test/baseline; **Post:** the neuroimaging findings and/or behavioral performance during post-test/after intervention; **L**: left; **R**: Right; **Bil**: bilateral; **amp**: amplitude; ↑: improved performance; ≈ : no significant changes between groups, or between pre and post-tests.

**Table S6**

| **Study, year** | **A/C** | **Variable** | **Within group (Pre vs. Post)** | | | **Between group (E vs C)** | | |
| --- | --- | --- | --- | --- | --- | --- | --- | --- |
|  |  |  | **Hedges’ g** | **95% CI (LL)** | **95% CI (UL)** | **Hedges’ g** | **95% CI (LL)** | **95% CI (UL)** |
| **SLEEPING QUALITY** | | | | | | | | |
| Bra 2015 | C | Total sleep time | -0.12 | -0.83 | 0.59 |  | | |
|  |  | Sleep efficiency | 0.75 | -0.08 | 1.57 |  |  |  |
|  |  | Sleep latency | 0.18 | -0.53 | 0.89 |  |  |  |
|  |  | # of awakenings | -0.09 | -0.80 | 0.61 |  |  |  |
|  |  | Awake time | -0.43 | -1.18 | 0.31 |  |  |  |
|  |  | Light sleep time | 0.08 | -0.62 | 0.78 |  |  |  |
|  |  | Light sleep (%) | -0.04 | -0.74 | 0.66 |  |  |  |
|  |  | Deep sleep time | 0.57 | -0.21 | 1.34 |  |  |  |
|  |  | Deep sleep (%) | 0.25 | -0.47 | 0.96 |  |  |  |
|  |  | REM sleep time | -0.42 | -1.16 | 0.33 |  |  |  |
|  |  | REM sleep (%) | -0.73 | -1.55 | 0.09 |  |  |  |
|  | **A** | Total sleep time | -0.15 | -0.85 | 0.56 |  | | |
|  |  | Sleep efficiency | 0.75 | -0.08 | 1.57 |  |  |  |
|  |  | Sleep latency | -0.23 | -0.95 | 0.48 |  |  |  |
|  |  | # of awakenings | -1.39 | -2.47 | -0.32 |  |  |  |
|  |  | Awake time | -0.78 | -1.61 | 0.06 |  |  |  |
|  |  | Light sleep time | -0.16 | -0.87 | 0.55 |  |  |  |
|  |  | Light sleep (%) | -0.37 | -1.11 | 0.37 |  |  |  |
|  |  | Deep sleep time | 0.20 | -0.52 | 0.91 |  |  |  |
|  |  | Deep sleep (%) | 0.46 | -0.30 | 1.21 |  |  |  |
|  |  | REM sleep time | 0.30 | -0.43 | 1.02 |  |  |  |
|  |  | REM sleep (%) | 0.43 | -0.31 | 1.18 |  |  |  |
| **EMOTIONAL RESPONSES** | | | | | | | | |
| Che 2016 | A | Frontal alpha asymmetry | -0.26 | -0.90 | 0.38 |  | | |
| Chu 2021 | A | **E1**- Frontal alpha asymmetry | 0.78 | 0.11 | 1.45 | 1.17 | 0.41 | 1.94 |
|  |  | **E2-** Frontal alpha asymmetry | -0.41 | -0.99 | 0.16 | -0.02 | -0.72 | 0.67 |
| Che 2021 | C | Frontal alpha asymmetry | 0.59 | -0.04 | 1.21 | -0.66 | -1.79 | 0.47 |
| **CORTICAL AROUSAL** | | | | | | | | |
| Hua 2017 | C | Theta @ F2, F4, Fz | -0.20 | -0.76 | 0.35 | -0.14 | -0.87 | 0.59 |
|  |  | Theta @ C3, C4,Cz | -0.03 | -0.57 | 0.52 | 0.15 | -0.58 | 0.88 |
|  |  | Theta @ P3, P4, Pz | 0.15 | -0.40 | 0.70 | 0.31 | -0.42 | 1.04 |
| Hua 2018 | A | Theta @ Fz | 0.09 | -0.33 | 0.51 | -0.08 | -0.64 | 0.49 |
|  |  | Theta @ Cz | 0.19 | -0.23 | 0.62 | 0.08 | -0.49 | 0.64 |
|  |  | Theta @ Pz | 0.28 | -0.15 | 0.71 | 0.11 | -0.46 | 0.68 |
| Lee 2017 | C | Beta wave @ F3 (EC) | 1.06 | -0.42 | 2.53 | 1.41 | 0.14 | 2.67 |
|  |  | Beta wave @ F3 (EO) | 0.73 | -0.53 | 1.99 | 0.56 | -0.60 | 1.71 |
|  |  | Beta wave @ F3 (Stroop) | 1.77 | -0.27 | 3.81 | 1.66 | 0.35 | 2.97 |
|  |  | Beta wave @ F4 (EC) | 0.40 | -0.70 | 1.51 | 0.74 | -0.43 | 1.91 |
|  |  | Beta wave @ F4 (EO) | 0.79 | -0.51 | 2.09 | 0.85 | -0.33 | 2.03 |
|  |  | Beta wave @ F3 (Stroop) | 0.70 | -0.55 | 1.94 | -0.49 | -1.64 | 0.66 |
| Hua 2018 | A | Beta @ Fz | 1.06 | -0.48 | 0.36 | -0.06 | -0.63 | 0.50 |
|  |  | Beta @ Cz | 0.10 | -0.32 | 0.52 | 0.10 | -0.46 | 0.67 |
|  |  | Beta @ Pz | 0.10 | -0.32 | 0.52 | 0.03 | -0.53 | 0.60 |
| Hua 2018 | A | Alpha @ Fz | 0.00 | -0.42 | 0.42 | -0.08 | -0.65 | 0.49 |
|  |  | Alpha @ Cz | 0.14 | -0.28 | 0.56 | 0.04 | -0.52 | 0.61 |
|  |  | Alpha @ Pz | -0.01 | -0.43 | 0.41 | -0.19 | -0.75 | 0.38 |
| Tsa 2021 | A | **E1**: Alpha @ Fz, Cz, Pz | 0.29 | -0.13 | 0.71 |  | | |
|  |  | **E2**: Alpha @ Fz, Cz, Pz | 0.20 | -0.22 | 0.61 |  |  |  |
|  |  | **E3**: Alpha @ Fz, Cz, Pz | -0.11 | -0.52 | 0.30 |  |  |  |
| Hua 2017 | C | Theta/alpha  @ F2, F4, Fz | -0.63 | -1.24 | -0.02 | -0.60 | -1.34 | 0.15 |
|  |  | Theta/alpha  @ C3, C4, Cz | -0.72 | -1.35 | -0.09 | -0.61 | -1.35 | 0.14 |
|  |  | Theta/alpha  @ P3, P4, Pz | -0.44 | -1.01 | 0.14 | -0.50 | -1.24 | 0.24 |
| Hua 2018 | A | Theta/alpha @ Fz | -0.05 | -0.47 | 0.36 | -0.16 | -0.73 | 0.41 |
|  |  | Theta/alpha @ Cz | -0.22 | -0.64 | 0.21 | -0.24 | -0.80 | 0.33 |
|  |  | Theta/alpha @ Pz | -0.04 | -0.46 | 0.38 | -0.05 | -0.62 | 0.51 |
| Hua 2017 | C | Theta/beta  @ C3, C4, Cz | 0.04 | -0.51 | 0.58 | 0.34 | -0.40 | 1.07 |
|  |  | Theta/beta  @ P3, P4, Pz | 0.60 | 0.00 | 1.21 | -0.61 | -1.35 | 0.14 |
|  |  | Theta/beta  @ F2, F4, Fz | 0.18 | -0.38 | 0.73 | 0.45 | -0.29 | 1.19 |
| Hua 2018 | A | Theta/beta @ Fz | -0.04 | -0.46 | 0.38 | -0.21 | -0.78 | 0.36 |
|  |  | Theta/beta @ Cz | -0.20 | -0.63 | 0.22 | -0.33 | -0.90 | 0.24 |
|  |  | Theta/beta @ Pz | -0.19 | -0.61 | 0.24 | -0.35 | -0.92 | 0.22 |
| **SOCIAL BRAIN CONNECTIVITY** | | | | | | | | |
| Cai 2020 | C | FA: Corpus callosum | -2.32 | -3.46 | -1.19 | -2.14 | -3.06 | -1.23 |
|  |  | FA: Fornix | 2.70 | 1.42 | 3.98 | 2.87 | 1.83 | 3.90 |
|  |  | FA: R cerebral peduncle | 1.07 | 0.35 | 1.78 | 1.22 | 0.43 | 2.02 |
|  |  | FA: L posterior limb of internal capsule | 0.53 | -0.06 | 1.12 | 0.65 | -0.10 | 1.39 |
|  |  | FA: R retrolenticular part of internal capsule | 0.46 | -0.12 | 1.04 | 0.67 | -0.08 | 1.42 |
|  |  | FA: L anterior corona radiate | 0.74 | 0.11 | 1.38 | 0.82 | 0.06 | 1.58 |
|  |  | FA: L superior fronto-occipital fasciculus | 0.75 | 0.11 | 1.38 | 1.07 | 0.29 | 1.85 |
|  |  | MD: L Corticospinal tract | -0.58 | -1.18 | 0.02 | -1.12 | -1.90 | -0.34 |
|  |  | MD: R Corticospinal tract | -0.72 | -1.35 | -0.09 | -1.51 | -2.34 | -0.69 |
|  |  | MD: L anterior corona radiate | -0.76 | -1.40 | -0.13 | -0.77 | -1.53 | -0.02 |
| EXECUTIVE FUNCTIONS | | | | | | | | |
| Jan 2016a | C | P3b amp @ Fz (success) | -0.31 | -0.74 | 0.12 | -0.78 | -1.36 | -0.20 |
|  |  | P3b amp @ Fz (fail) | -0.23 | -0.66 | 0.19 | -0.84 | -1.42 | -0.25 |
|  |  | P3b amp @ Cz (success) | -0.22 | -0.64 | 0.21 | -1.08 | -1.68 | -0.48 |
|  |  | P3b amp @ Cz (fail) | -0.18 | -0.60 | 0.25 | -1.21 | -1.82 | -0.60 |
|  |  | P3b amp @ Pz (success) | -0.30 | -0.73 | 0.13 | -0.89 | -1.48 | -0.30 |
|  |  | P3b amp @ Pz (fail) | -0.28 | -0.71 | 0.15 | -1.11 | -1.71 | -0.51 |
| Smi 2019 | C | P3b amp @ parietal (Go) | -0.54 | -1.19 | 0.11 | -0.22 | -0.95 | 0.52 |
|  |  | P3b amp @ parietal (No Go) | -0.34 | -0.96 | 0.28 | -0.13 | -0.87 | 0.60 |
| Mil 2019 | C | P3b amp @ Cz (SA) | 0.41 | 0.07 | 0.76 | 0.60 | 0.10 | 1.09 |
|  |  | P3b amp @ fCz (CON) | 0.31 | -0.03 | 0.65 | 0.39 | -0.10 | 0.88 |
|  |  | P3b amp @ fCz (IN) | 0.43 | 0.08 | 0.78 | 0.48 | -0.01 | 0.98 |
| Tsa 2014 | C | P3b amp @ Pz |  | | | 1.13 | 0.46 | 1.80 |
| Lud 2017 | A | P3b amp @ parietal (Aerobic) | 0.84 | 0.21 | 1.47 | 0.84 | 0.12 | 1.56 |
|  |  | P3b amp @ parietal  (Coordination) | 0.06 | -0.47 | 0.59 | 0.06 | -0.63 | 0.75 |
| Yu 2020 | A | P3b amp @ CPz, Pz (CON) |  | | | 0.10 | -0.47 | 0.66 |
|  |  | P3b amp @ CPz, Pz (IN) |  |  |  | -0.21 | -0.78 | 0.35 |
| Smi 2019 | C | P3b latency @ parietal (Go) | -0.30 | -0.91 | 0.31 | -1.24 | -2.04 | -0.44 |
|  |  | P3b latency @ parietal (No Go) | -0.15 | -0.75 | 0.45 | -0.03 | -0.76 | 0.70 |
| Mil 2019 | C | P3b latency @ Cz (SA) | -0.15 | -0.48 | 0.18 | -0.86 | -1.37 | -0.35 |
|  |  | P3b latency @ fCz (CON) | 0.19 | -0.14 | 0.53 | 0.00 | -0.48 | 0.49 |
|  |  | P3b latency @ fCz (IN) | 0.49 | 0.14 | 0.84 | 0.14 | -0.35 | 0.62 |
| Tsa 2012 | C | P3 latency @ IPS, STS |  | | | -1.26 | -2.04 | -0.47 |
| Lud 2017 | A | P3b latency @ parietal (Aerobic) | -0.25 | -0.78 | 0.29 | -0.48 | -1.18 | 0.23 |
|  |  | P3b latency @ parietal (Coordination) | -0.06 | -0.59 | 0.47 | -0.29 | -0.98 | 0.41 |
| Yu 2020 | A | P3b latency @ CPz, Pz (CON) |  | | | 0.21 | -0.35 | 0.78 |
|  |  | P3b latency @ CPz, Pz (IN) |  |  |  | 0.19 | -0.37 | 0.76 |
| Smi 2019 | C | N2 amp @ frontal central (Go) | 0.34 | -0.28 | 0.95 | 0.14 | -0.59 | 0.88 |
|  |  | N2 amp @ frontal central (No Go) | -0.23 | -0.83 | 0.38 | -0.16 | -0.89 | 0.57 |
| Jan 2016a | C | N2 amp @ Fz (success) | 0.02 | -0.40 | 0.44 | -0.07 | -0.63 | 0.49 |
|  |  | N2 amp @ Fz (fail) | -0.39 | -0.83 | 0.05 | -0.06 | -0.62 | 0.50 |
|  |  | N2 amp @ Cz (success) | -0.33 | -0.76 | 0.11 | -0.46 | -1.03 | 0.11 |
|  |  | N2 amp @ Cz (fail) | -0.39 | -0.83 | 0.05 | -0.30 | -0.86 | 0.26 |
|  |  | N2 amp @ Pz (success) | -0.38 | -0.82 | 0.06 | -0.52 | -1.09 | 0.05 |
|  |  | N2 amp @ Pz (fail) | -0.39 | -0.82 | 0.05 | -0.46 | -1.03 | 0.11 |
| Yu 2020 | A | N2 amp @ Fz, FCz, Cz (CON) |  | | | -0.22 | -0.79 | 0.34 |
|  |  | N2 amp @ Fz, FCz, Cz (IN) |  |  |  | -0.36 | -0.93 | 0.21 |
| Vog 2013 | A | N2 amp @ occipital | 0.05 | -0.61 | 0.71 | 0.29 | -0.55 | 1.13 |
| Smi 2019 | C | N2 latency @ frontal central (Go) | -0.36 | -0.98 | 0.26 | -0.30 | -1.04 | 0.44 |
|  |  | N2 latency @ frontal central (No Go) | -0.29 | -0.90 | 0.32 | 0.10 | -0.63 | 0.84 |
| Yu 2020 | A | N2 latency @ Fz, FCz, Cz (CON) |  | | | -0.45 | -1.02 | 0.12 |
|  |  | N2 latency @ Fz, FCz, Cz (IN) |  |  |  | -0.18 | -0.75 | 0.39 |
| Bre 2020 | A | Hb-Oxy @ prefrontal (Circuit) | 0.64 | -0.06 | 1.35 |  | | |
|  |  | Hb-Oxy @ prefrontal (treadmill) | 0.10 | -0.53 | 0.73 |  |  |  |
| Cho 2015 | C | BOLD @ R frontal | 1.51 | 0.58 | 2.44 | 1.05 | 0.28 | 1.82 |
|  |  | BOLD @ L parietal | 1.10 | 0.31 | 1.89 | 0.66 | -0.08 | 1.40 |
|  |  | BOLD @ R temporal | -2.17 | -3.36 | -0.98 | -1.81 | -2.66 | -0.95 |
|  |  | BOLD @ R occipital | 0.14 | -0.46 | 0.74 | 0.05 | -0.68 | 0.77 |
|  |  | BOLD @ R parietal | 0.09 | -0.51 | 0.69 | 0.01 | -0.71 | 0.73 |
|  |  | BOLD @ R cerebellum | -0.12 | -0.72 | 0.48 | -0.02 | -0.75 | 0.70 |
|  |  | BOLD @ L temporal | -0.17 | -0.77 | 0.43 | -0.07 | -0.79 | 0.65 |
| OTHER VARIABLES | | | | | | | | |
| Lag, 2019 | C | P50 amp @ Cz (Conditional click) | -0.26 | -1.19 | 0.67 |  | | |
|  |  | P50 amp @ Cz  (test click) | -0.04 | -0.94 | 0.87 |  |  |  |
|  |  | P50 amp @ Cz  (Difference score) | -0.18 | -1.10 | 0.74 |  |  |  |
|  |  | N100 amp @ Cz (Conditional click) | -0.90 | -2.61 | 0.80 |  |  |  |
|  |  | N100 amp @ Cz  (test click) | 0.71 | -0.83 | 2.25 |  |  |  |
|  |  | N100 amp @ Cz  (Difference score) | -1.03 | -2.86 | 0.79 |  |  |  |
| Cor, 2016 | C | ERP @ parietal | 0.66 | 0.09 | 1.24 | 0.88 | 0.13 | 1.64 |
| Vog, 2012 | A | Current density @ Rectal gyrus | -0.98 | -1.83 | -0.13 |  | | |
|  |  | Current density @ medial frontal gyrus | -0.46 | -1.16 | 0.25 |  |  |  |
|  |  | Current density @ orbital gyrus | -1.11 | -2.01 | -0.22 |  |  |  |
|  |  | Current density @ Brodmann area 11 | -1.06 | -1.93 | -0.18 |  |  |  |
|  |  | Current density @ Brodmann area 25 | -0.73 | -1.50 | 0.04 |  |  |  |

A = Acute; C = Chronic; Amp = amplitude; LL= the lower limb of the 95% CI; UL = the upper limb of the 95% CI; E: experimental group (E1= the first experimental group; E2 = the second experimental group; E3 = the third experimental group); R = right; L = left; Bil = bilateral; EC = eye closed; EO = eye opened; success = the successful trials during the stop signal task; fail = the failed trials during the stop signal task; CON = the congruent condition during the Flanker task; IN = the incongruent condition during the Flanker task; Go = the Go condition during the Go-no-Go task; No-Go = the No go condition during the Go-no-Go task; ERP = event related potentials; BOLD = Blood oxygenation level-dependent; Oxy-Hb = oxyhemoglobin; Shaded variable indicates that the 95% CIs of the between and/or the within groups comparisons doesn’t include 0. Note that to ensure accuracy and to allow between-study comparisons, this table only includes the effect sizes of the outcome variables for which the means, standard deviation/standard error of means, and study sample sizes were provided by the original papers.
